# Estimating Volumes of Near-Spherical Molded Artifacts

**DOI:** 10.6028/jres.115.009

**Published:** 2010-06-01

**Authors:** David E. Gilsinn, Bruce R. Borchardt, Amelia Tebbe

**Affiliations:** Mathematical and Computational Sciences Division, Information Technology Laboratory, National Institute of Standards and Technology, Gaithersburg, MD 20899-8910; Precision Engineering Division, Manufacturing Engineering Laboratory, National Institute of Standards and Technology, Gaithersburg, MD 20899-8211; Department of Mathematics, St. Mary’s College of Maryland, St. Mary’s City, MD 20686-3001

**Keywords:** B-splines, computed tomography, coordinate measuring machine, divergence theorem, lung cancer, lung cancer phantoms, nonlinear least squares

## Abstract

The Food and Drug Administration (FDA) is conducting research on developing reference lung cancer lesions, called phantoms, to test computed tomography (CT) scanners and their software. FDA loaned two semi-spherical phantoms to the National Institute of Standards and Technology (NIST), called Green and Pink, and asked to have the phantoms’ volumes estimated. This report describes in detail both the metrology and computational methods used to estimate the phantoms’ volumes. Three sets of coordinate measuring machine (CMM) measured data were produced. One set of data involved reference surface data measurements of a known calibrated metal sphere. The other two sets were measurements of the two FDA phantoms at two densities, called the coarse set and the dense set. Two computational approaches were applied to the data. In the first approach spherical models were fit to the calibrated sphere data and to the phantom data. The second approach was to model the data points on the boundaries of the spheres with surface B-splines and then use the Divergence Theorem to estimate the volumes. Fitting a B-spline model to the calibrated sphere data was done as a reference check on the algorithm performance. It gave assurance that the volumes estimated for the phantoms would be meaningful. The results for the coarse and dense data sets tended to predict the volumes as expected and the results did show that the Green phantom was very near spherical. This was confirmed by both computational methods. The spherical model did not fit the Pink phantom as well and the B-spline approach provided a better estimate of the volume in that case.

## 1. Introduction

Tomography is a method of imaging a single slice of the body. Modern computed tomography (CT) is a medical imaging method that uses tomography, but also employs digital image processing techniques, to generate three dimensional images built from a large sequence of two-dimensional x-ray images made around a single axis. CT has shown promising results in detecting lung cancers at more operable stages, when survival is better [1].

**Fig. 1:**
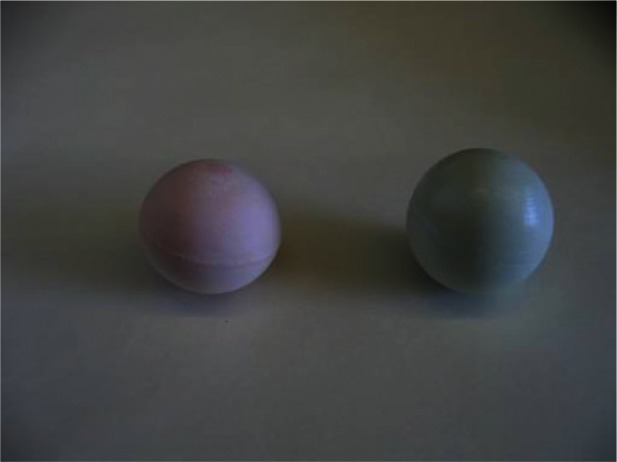
Two simulated lung cancers, called phantoms.

The Food and Drug Administration (FDA) is conducting research on developing reference cancer lesions, called phantoms, to test CTs and their software. Two samples were loaned to NIST to estimate volumes. The material of the phantoms simulates lung cancer material. The phantoms can be inserted into a simulated body torso for CT scans. The two phantoms are shown in Fig. 1. Although they seem spherical, they are slightly non-spherical. The larger one on the right is referred to as the Green phantom and the one on the left is called the Pink phantom due to the material colors.

One experimental approach to estimate the volume of the phantoms would be to use an Archimedes test in which the phantoms would be immersed in a liquid bath in a well calibrated container with fine measurement gradations to determine liquid displacement. However, in the case of these phantoms, the material used to manufacture these phantoms was porous and the phantoms would have to be coated. This, of course, would affect the “ground truth” volume estimate. As a result, the approach chosen for this study was based on a fundamental theorem in calculus, called the Divergence Theorem (see Taylor [2]), an analogue of Green’s Theorem in two dimensional space. In the Divergence Theorem a volume integral is shown to be equal to a surface integral. Therefore, we surmised that, if a model of the surfaces of the phantoms could be developed, the Divergence Theorem would help to estimate their volumes. In order to develop a surface model we needed to obtain data about the surfaces of the phantoms. This was done using a coordinate measuring machine (CMM) in the Manufacturing Engineering Laboratory (MEL) at NIST. This machine produced a set of (*x*, *y*, *z*) points on the surface of each phantom. The data were then transformed to spherical coordinates and modeled using a set of basis functions, called B-splines. After fitting, the B-spline model was employed to generate a grid of values on the surface. These values were used to form surface triangles that were then used to compute the necessary surface integrals and finally the volume. The quality of the volume estimates depended on the surface grid sizes, as will be clear from the discussion below. The method of B-spline surface modeling is not new, in that it was suggested in the book by Dierckx [3]. However the application in the current case, that uses the Divergence Theorem, appears to be new. A reader can also consult the Dierckx references [4] and [5].

The paper is divided as follows. Section 2 describes how a volume can be computed using the Divergence Theorem. Section 3 describes the surface point generation experiments using the CMM. Section 4 introduces the two methods of data modeling used to estimate the phantoms’ volumes. The first uses a spherical model. The second uses the B-splines as a basis for a least squares fit with regularization to model the phantoms’ surface data. The fitted functions were then used to generate the grid data necessary to apply the Divergence Theorem. Section 5 presents the specific surface triangulation used to implement the Divergence Theorem. Section 6 describes the volumetric results from applying the spherical and B-spline models to the CMM data. A summary is given in Sec. 7.

## 2. Volume Estimation by the Divergence Theorem

In this section we state the Divergence Theorem and indicate how it can be used to estimate the volume of a polyhedron. This provides the motivation for the need to determine surface data points from the phantoms and then to build a surface model that is used to create data points at surface triangulation vertices. As the number of triangles was increased, the volume estimates were expected to tend to a fixed value. As will be seen, this expectation is confirmed below.

### 2.1 Divergence Theorem in 3-D and Volume Computation

Let Ω be a simply connected region in three dimensional space and Γ the surface boundary. Then

(1) $$\int \int_{\Omega }\int \vec{\nabla }\cdot F\;dxdydz=\int_{\Gamma }\int F\cdot \hat{n}\;d\sigma ,$$

where *F* (*x*, *y*, *z*) = (*F*_1_ (*x*, *y*, *z*), *F*_2_ (*x*, *y*, *z*), *F*_3_ (*x*, *y*, *z*)) is a differentiable vector field,

(2) $$\vec{\nabla }\cdot F=\frac{\partial F_{1}}{\partial x}+\frac{\partial F_{2}}{\partial y}+\frac{\partial F_{3}}{\partial z},$$

is the divergence of the vector field, $\hat{n}$ is an outward-pointing unit normal vector to Γ, and *dσ* is an infinitesimal element of the surface.

If we select *F* (*x*, *y, z*) = (1/3)(*x, y z*), then $\vec{\nabla }\cdot F=1$ and we can write

(3) $$\int \int_{\Omega }\int dxdydz=\frac{1}{3}\int_{\Gamma }\int \hat{n}\cdot (x,y,z)\;d\sigma .$$

Note that the left hand side is simply the volume of the region Ω. The 1/3 factor comes from the definition of *F* (*x*, *y, z*) so that $\vec{\nabla }\cdot F=1$. Now, if Γ is approximated by disjoint polyhedra (planar surface patches), Γ*_i_*, then

(4) $$\Gamma =\overset{N}{\underset{i=1}{\cup }}\Gamma_{i},$$

and

(5) $$\frac{1}{3}\int_{\Gamma }\int \hat{n}\cdot (x,y,z)\;d\sigma =\frac{1}{3}\sum_{i=1}^{N}\int_{\Gamma_{i}}\int {\hat{n}}_{i}\cdot (x,y,z)\;d\sigma ,$$

where $\hat{n}$ is the unit outer normal to Γ*_i_*. Here we will model the surface patches by planar facets and, in particular, triangular facets. The plane for Γ*_i_* is given by ${\hat{n}}_{i}\cdot (x,y,z)=c_{i}$, where *c_i_* is a constant associated with each triangular facet. The sum of the integrals over the facets thus reduces to

(6) $$\frac{1}{3}\sum_{i=1}^{N}\int_{\Gamma_{i}}\int {\hat{n}}_{i}\cdot (x,y,z)d\sigma =\frac{1}{3}\sum_{i=1}^{N}\int_{\Gamma_{i}}\int c_{i}\;d\sigma =\frac{1}{3}\sum_{i=1}^{N}c_{i}\;\text{Area}(\Gamma_{i}).$$

This method of computing the volume of an object from a surface integral can be found in Schneider and Eberly [6].

### 2.2 Area of a Planar Polyhedron in 3-D

In order to estimate the surface area of a facet, as used in (6), we will describe the process of computing the area of a planar polyhedron in space by use of Stokes’ Theorem and then we will particularize it to a planar triangle in space. Here the assumption is made that there is surface data available at the planar triangle vertices. Stokes’ Theorem states that if *C* is a piecewise smooth boundary curve, oriented positively, of a surface Γ, and if *F* is a differentiable vector field defined on Γ and $\hat{n}$ is a unit normal satisfying the right-hand rule relative to the boundary orientation, then

(7) $$\int_{\Gamma }\int \left(\vec{\nabla }\times F\right)\cdot \hat{n}\;d\sigma =\int_{C}F\cdot d\vec{R},$$

where the curl of *F*(*x, y, z*) = (*F*_1_ (*x, y, z*), *F*_2_(*x, y, z*), *F*_3_(*x, y, z*)) is

(8) $$\vec{\nabla }\times F=\left(\frac{\partial F_{3}}{\partial y}-\frac{\partial F_{2}}{\partial z},\frac{\partial F_{1}}{\partial z}-\frac{\partial F_{3}}{\partial x},\frac{\partial F_{2}}{\partial x}-\frac{\partial F_{1}}{\partial y}\right),$$

and $d\vec{R}=(dx,dy,dz)$. If we set *F*(*x, y, z*) = (1/2) $\hat{n}\times (x,y,z)$ then $\vec{\nabla }\times F=\hat{n}$ and $\hat{n}\cdot \hat{n}=1$ so that

(9) $$\begin{array}{c} \text{Area}\;(\Gamma )=\int_{\Gamma }\int d\sigma =\frac{1}{2}\oint_{C}\left(\hat{n}\;\times (x,y,z)\right)\cdot (dx,dy,dz),\\ \;=\frac{1}{2}\oint_{C}\left(\hat{n}\cdot ((x,y,z\right)\times (dx,dy,dz)),\\ \;=\frac{1}{2}\oint_{C}\hat{n}\cdot ((x(t),y(t),z(t))\times (x^{\prime }(t),y^{\prime }(t),z^{\prime }(t)))\;dt,\\ \;=\frac{1}{2}\oint_{C}\hat{n}\cdot (y(t)z^{\prime }(t)-z(t)y^{\prime }(t),z(t)x^{\prime }(t)-x(t)z^{\prime }(t),x(t)y^{\prime }(t)-y(t)x^{\prime }(t))\;dt. \end{array}$$

The factor 1/2 comes from the definition of *F*(*x, y, z*) so that $(\vec{\nabla }\times F)=\hat{n}$.

We will now specialize the Stokes formula to find the area of a spatial triangle, γ, in terms of its three vertices oriented positively. Let the three vertices, in positive orientation, be specified by *v_1_* = (*x*_1_,*y*_1_,*z*_1_), *v_2_* = (*x*_2_, *y*_2_, *z*_2_), *v*_3_ = (*x*_3_, *y*_3_, *z*_3_). Parameterize the boundary of the triangle as follows. For *t* ∈ [0, 1], let *v* = *v*_1_ + *t*(*v*_2_ *−v*_1_) = (*x*_1_ + *t*(*x_2_ −x*_1_), *y*_1_ + *t*(*y*_2_ *−y*_1_), *z*_1_ + *t*(*z*_2_ *−z*_1_)). For *t* ∈[1, 2] let *v* = *v*_2_ + (*t* −1) (*v*_3_ −*v*_2_) = (*x*_2_ + (*t* −1)(*x*_3_ −*x*_2_), *y_2_ +* (*t −*1)(*y*_3_ *−y_2_*), *z*_2_ + (*t* −1)(*z*_3_ −*z*_2_)). Finally, for *t* ∈ [2, 3], put *v = v_3_ +* (*t −*2)(*v*_1_ −*v*_3_) = (*x*_3_ + (*t* −2)(*x*_1_ −*x*_3_), *y_3_ +* (*t −2*)(*y*_1_ *−y*_3_), *z_3_ +* (*t −*2)(*z*_1_ −*z*_3_)). The area of the spatial triangle, in terms of the parameterized boundary, can be written as

(10) $$\text{Area}\;(\gamma )=\frac{1}{2}\oint_{C}\hat{n}\cdot (y(t)z^{\prime }(t)-z(t)y^{\prime }(t),\;z(t)x^{\prime }(t)-x(t)z^{\prime }(t),\;x(t)y^{\prime }(t)-y(t)x^{\prime }(t))\;dt,\;=\frac{1}{2}\hat{n}\cdot \left(\int_{0}^{3}(y(t)z^{\prime }(t)-z(t)y^{\prime }(t))\;dt,\;\int_{0}^{3}\left(z(t)x^{\prime }(t\right)-x(t)z^{\prime }(t))\;dt,\;\int_{0}^{3}\left(x(t)y^{\prime }(t\right)-y(t)x^{\prime }(t))\;dt\right)$$

By straightforward integration over each of the parameterized segments it is easy to show that

(11) $$\begin{array}{c} \text{Area}\;(\gamma )=\frac{1}{2}\hat{n}\cdot ((y_{1}z_{2}-y_{2}z_{1})+(y_{2}z_{3}-y_{3}z_{2})+(y_{3}z_{1}-y_{1}z_{3}),\\ \;(z_{1}x_{2}-z_{2}x_{1})+(z_{2}x_{3}-z_{3}x_{2})+(z_{3}x_{1}-z_{1}x_{3}),\\ \;(x_{1}y_{2}-x_{2}y_{1})+(x_{2}y_{3}-x_{3}y_{2})+(x_{3}y_{1}-x_{1}y_{3})). \end{array}$$

The normal vector to the oriented triangle can be computed as follows. Let $\vec{v}=(x_{2}-x_{1},\;y_{2}-y_{1},\;z_{2}-z_{1})$ and $\vec{w}=(x_{3}-x_{1},\;y_{3}-y_{1},\;z_{3}-z_{1})$. Then $\vec{n}=\vec{v}\times \vec{w}=((y_{2}-y_{1})(z_{3}-z_{1})-(z_{2}-z_{1})(y_{3}-y_{1}),\;(z_{2}-z_{1})(x_{3}-x_{1})-(x_{2}-x_{1})(z_{3}-z_{1}),\;(x_{2}-x_{1})(y_{3}-y_{1})-(y_{2}-y_{1})(x_{3}-x_{1}))$. If we set *n_1_* = (*y*_2_ *−y*_1_)(*z*_3_ *−z*_1_)*−*(*z*_2_ *−z*_1_)(*y*_3_ *−y*_1_), *n*_2_ = (*z*_2_ *−z*_1_)(*x*_3_ *−x*_1_) *−*(*x*_2_ *−x*_1_)(*z*_3_ *−z*_1_), *n_3_* = (*x*_2_ *−x*_1_)(*y −y*_1_) *−*(y_2_ *−y*_1_)(*x*_3_ *−x*_1_), then $\left\|\vec{n}\right\|=\sqrt{n_{1}^{2}+n_{2}^{2}+n_{3}^{2}}$ and $\hat{n}=\vec{n}/\left\|\vec{n}\right\|$.

## 3. Surface Metrology of the Artifacts

**Fig. 2:**
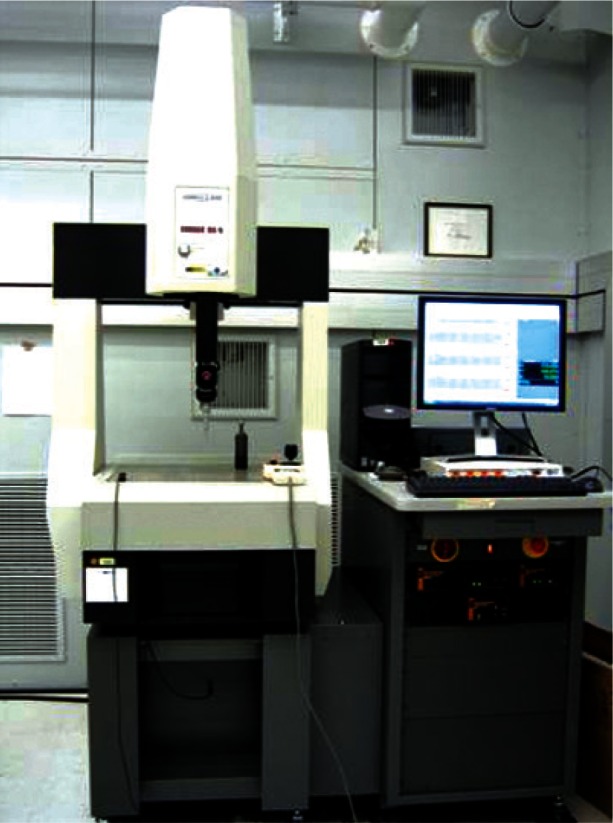
Computer controlled CMM.

In this section we discuss the experimental method used to obtain surface data for the three objects used in this study. As a test object for the modeling process, a well calibrated sphere was selected. This object, along with the FDA phantoms formed the study artifact set. Data points, in the form of (*x, y, z*) coordinates, were created by the probing action of the CMM. Figure 2 shows the CMM system used to measure the artifacts. The system is computer controlled and touches an object to be measured at programmed points in order to produce (*x, y, z*) values at the probed points.

**Fig. 3:**
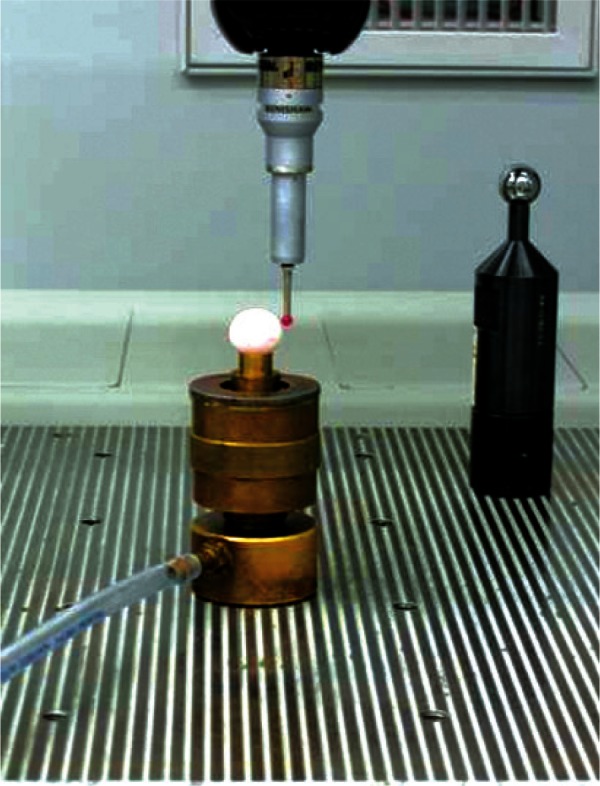
Lung nodule phantom on the vacuum chuck.

Figure 3 shows one of the phantoms, held by a device called a vacuum chuck, as it is being touched by the CMM probe. The probe itself can be programmed to approach an object at various angles. In the background of the figure is a high quality reference steel sphere. Before an object is probed the reference sphere is measured to determine the effective diameter of the CMM probe tip. This reference sphere is separate from the calibrated sphere used to test the modeling process. The difference between the known diameter of the reference steel sphere and the apparent diameter of the measured reference metal sphere gave the calibrated effective probe diameter.

The FDA phantoms were created by a molding process and the mold marks on both spheres were visible. These mold marks were used to align the phantoms with the coordinate system of the CMM. The marks were laid out like lines of latitude, leading to the use of a natural nomenclature of latitude and longitude like those of the Earth. For each phantom, the North Pole was chosen to be the one with darker, deeper, or more obvious mold marks.

For the purpose of understanding the measurement process we will describe the physical fixture positioning of the phantoms. They were held by a vacuum chuck (see Fig. 3) to minimize distortion and reduce the chance of damaging the spheres’ surfaces. As Fig. 3 shows, the chuck has a shallow cone to hold the phantoms. The phantoms contacted the cone around a circle of latitude at about −45°. This was measured from the equatorial circle around the middle of the phantoms. For example, the point at the top of the phantoms would be at + 90°. The wall vacuum of the cone was strong enough to hold the spheres sufficiently that they did not move significantly, as shown by the repeatable results from run to run at the 1 μm level. The setup of the phantoms in the vacuum chuck was accomplished by eye alignment using the visible mold marks and minor imperfections as guides to the eye. The expanded uncertainty of alignment was estimated to be approximately ± 2° for the vertical angle (i.e., keeping the equator horizontal) and ± 4° for the azimuthal angle. For a presentation of the guidelines for how expanded uncertainty of parameters is computed see Taylor and Kuyatt [7]. Essentially, the guideline for estimating the expanded uncertainty involves applying a multiplier, *k* = 2, times the square root of the variance about a predicted parameter value (i.e., two times the standard error). For a full discussion of confidence intervals for parameter estimation see Draper and Smith [8].

Three sets of measurements were planned for each of the phantoms. In each, points were probed on only a hemisphere at a time and later the data sets were post-processed to form a spherical data set. In the first set of measurements the North Pole was set as the top point. A set of points was programmed to probe the top hemisphere down to the equator. The phantom was then re-positioned in the chuck so that the South pole was the top point. The same hemispherical points were probed. The rotation was done in such a manner that the mold marks representing the longitudinal lines were kept as aligned as possible. Post-processing of the data associated the correct signs with the measured coordinates relative to the CMM coordinate system. The third measurement involved re-positioning the phantoms so that the equatorial circle was vertical and the North-South axis was horizontal. Again two hemisphere sets of probe points were measured. This position was not feasible for the Pink phantom in one of the experiments described below.

Visually, the pink sphere was noticeably out-of-round, in the shape of an oblate spheroid. A dial caliper gave diameter measurements given in Table 1. The uncertainty of caliper measurements on hard steel surfaces is about 0.1 mm, and is estimated to be about 0.3 mm on the sample spheres due to the potential that the contact force would distort the soft surfaces of the spheres. All estimated uncertainties were *k* = 2 expanded uncertainties.

**Fig. 4:**
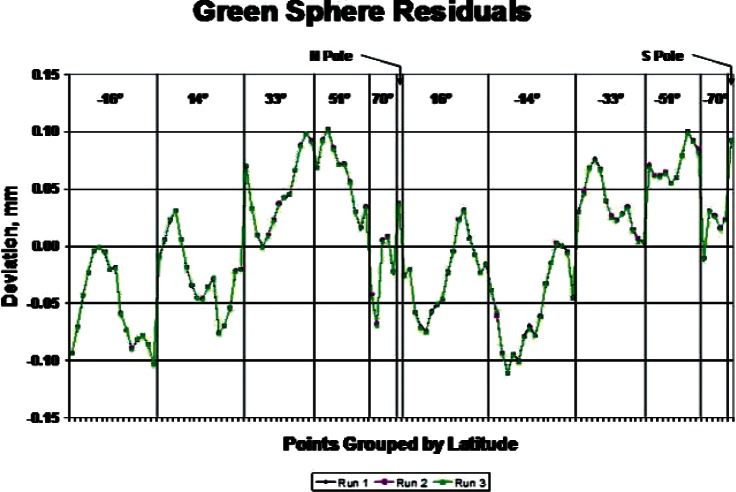
Residual Measures CMM Errors for the Green Phantom Sphere.

**Fig. 5:**
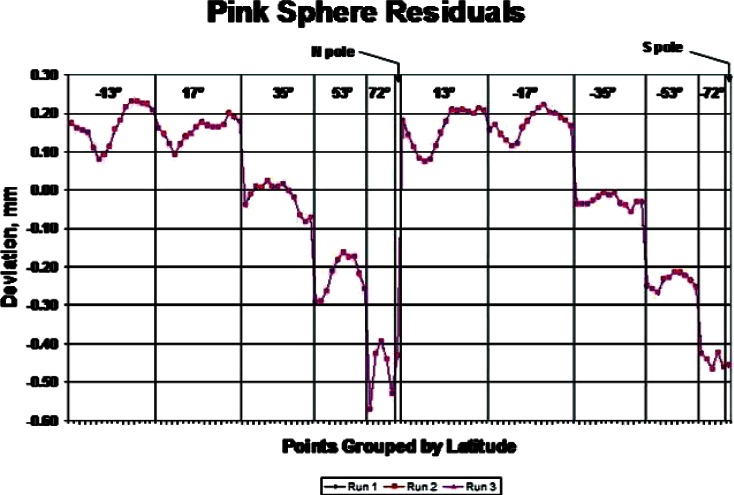
Residual Measures CMM Errors for the Pink Phantom Sphere.

**Fig. 6:**
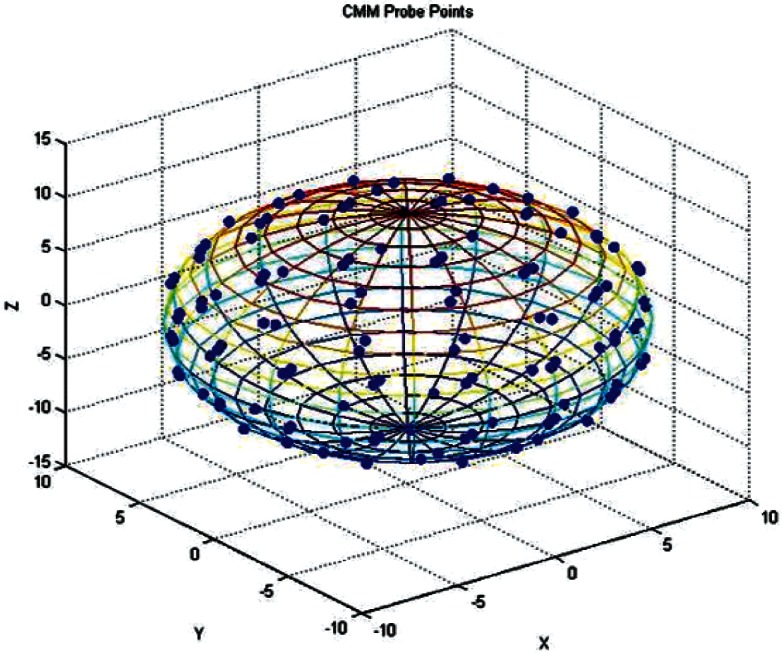
Sample Distribution of the CMM Probe Points on a Sphere.

Two probing experiments were performed on each of the phantoms and the calibrated sphere. They created what we will call a coarse data set and a dense data set. For the coarse data set the plan was to measure each phantom on the CMM three times in each position, with 61 coordinate points per hemisphere. For the green sphere, each measurement set consisted of three separate sets of points: North pole up, South pole up, and prime meridian/equator intersection up. The pink sphere could not be held sideways in the first experiment, as the out-of-roundness prevented an effective vacuum seal. Therefore, a measurement data set for this sphere had only the North Pole up and South Pole up data. The third data set in this case was a re-measure of the North Pole up position. Each data set consisted of 122 points. The plots in Fig. 4 and 5 show the radial deviation from a best-fit sphere for the full data sets. The figures show the radial residuals obtained by fitting sphere models to the Green and Pink data with an algorithm ordinarily used during sphere calibration work. In particular, they represent the residual errors between the distance from the fitted sphere center to the probed points and the fitted radius of the sphere model. The residuals, in the case of the Green phantom, range from approximately −0.1 mm to + 0.1 mm, whereas the residuals, in the case of the Pink phantom, range from approximately −0.57 mm to + 0.23 mm. This suggests the slight non-spherical nature of the Pink phantom. Figure 6 shows the typical distribution of the probe points on a sphere. The plot is a transparency so that probe points on the opposite side of the sphere are visible. The calibrated sphere on the shaft, with a diameter of 19.05 mm, was also measured with 122 points, repeated five times. It was measured in one position, since it was permanently mounted on a support shaft.

For the dense data set 181 points were taken on the phantoms and the calibrated sphere, with five repeats in each position. The positions were taken the same as those in the first experiment. That is, the alignment was selected with North pole up (position 1), South pole up (position 2), and prime meridian/equator intersection up (position 3). In this case it was possible to hold the Pink phantom in the sideways position 3. The calibrated sphere was also measured at 181 points with five repeats.

## 4. Modeling Methodologies

In this section two forms of data modeling will be discussed. Since the phantoms seemed to be nearly spherical the natural tendency was first to consider fitting a spherical model to each phantom and estimating the volume of the fitted spheres. However, in order to develop a potentially more accurate volume estimation model, the surface data was also fit using tensor products of B-splines and the volumes estimated by the Divergence Theorem.

### 4.1 A Spherical Model

In this section we consider how close the data could be modeled by a spherical model for the data. The calibrated sphere, of course, could be modeled via a spherical model.

In particular, let *c* = (*c*_1_, *c*_2_, *c*_3_, *c*_4_) and set

(12) $$F(x,y,z,c)={\left(x-c_{1}\right)}^{2}+{\left(y-c_{2}\right)}^{2}+{\left(z-c_{3}\right)}^{2}-c_{4}{}^{2}.$$

The unknown parameters *c*_1_, *c*_2_, *c*_3_ represent the center of the sphere and *c*_4_ is the radius. All of the data were measured in millimeters so that the parameters naturally have millimeter units. Define

(13) $$O(c)={\sum_{i=1}^{n}\left\{{\left(x_{i}-c_{1}\right)}^{2}+{\left(y_{i}-c_{2}\right)}^{2}+{\left(z_{i}-c_{3}\right)}^{2}-c_{4}{}^{2}\right\}}^{2},$$

where *O*, the objective function, is a measure of the residual for the fitted sphere defined by the coefficients *c*. Since the function *O* is a nonlinear implicit function of the parameters we needed to use a nonlinear minimization algorithm to find the best fit, i.e., to solve the problem

(14) $$\underset{c}{\min}\;O(c)\cdot$$

There are a number of algorithms for fitting least squares models to data on geometric shapes (see Shakarji [9]). There are also various algorithms for minimizing general nonlinear functions, such as (13). All of the algorithms involve iterative minimization of some form. Many require computing derivatives of the objective function in order to generate search directions along which to identify a minimum. Others do not involve derivatives but may be somewhat slower in the minimization search. The algorithm selected here, because of the relatively few parameters involved, and the fact that derivatives are not required, is a form of polyhedron search method called the Nelder-Mead method (see Sauer [10]). The Newton method, requiring derivatives, was initially used to estimate the parameters, but the Nelder-Mead tended to produce the smallest value to (13).

The Nelder-Mead algorithm works iteratively through steps that involve reflections, vertex extensions, and multidimensional polyhedron contractions until the volume of the polyhedron becomes less than a prescribed tolerance. The polyhedron vertices then enclose the minimum. The median value of the polyhedron vertex values is taken as the minimum value.

The uncertainties of the estimated center and radius were computed using the methods proposed in Draper and Smith [8] for nonlinear regression. In particular, if $\hat{c}=({\hat{c}}_{1},{\hat{c}}_{2},{\hat{c}}_{3},{\hat{c}}_{4})$, then define the *n* × 4 matrix with elements

(15) $${\hat{Z}}_{ij}=\frac{\partial F}{\partial c_{i}}(x_{i},y_{i},z_{i},\hat{c}),\;i=1,\cdots n,\;j=1,2,3,4.$$

The *i*-th row is given by

(16) $${\hat{Z}}_{i}=[-2(x_{i}-{\hat{c}}_{1})-2(y_{i}-{\hat{c}}_{2})-2(z_{i}-{\hat{c}}_{3})-2{\hat{c}}_{4}].$$

The standard error of ${\hat{c}}_{i}$, s.e. $\left({\hat{c}}_{i}\right)$, is given by the square root of the *i*-th diagonal element of ${\left({\hat{Z}}^{\prime }\hat{Z}\right)}^{-1}\;s^{2}$ where $s=O(\hat{c})/(n-4)$. The expanded uncertainty is given by 2s.e. $\left({\hat{c}}_{i}\right)$ as defined in Taylor and Kuyatt [20]. The uncertainty limits of ${\hat{c}}_{i}$ are ${\hat{c}}_{i}\pm 2s.e$. $\left({\hat{c}}_{i}\right)$ All units are in millimeters except volume, which is in cubic millimeters.

### 4.2 A B-spline Model

Given measured data points on the surfaces of the calibrated sphere and the two phantoms, surface models can be constructed using B-splines as basis functions. In this section we will define cubic B-splines and show the construction of tensor products of B-splines.

#### 4.2.1 Cubic B-Splines in One Variable

Suppose that a function *y = f*(*x*), *x* ***∈*** [*a*, *b*], is known at the *n* points (*x*_1_, *y*_1_),⋯, (*x_n_*, *y_n_*), where *a*<*x*_1_<*x*_2_< ⋯ <*x_n_<b*, *y_q_* =*f*(*x_q_*), *q* = 1,⋯, *n. a* and *b* are finite interval bounds. It is known that a polynomial of degree *n −*1, *P*(*x*), can be constructed to pass through these *n* points. In the case of highly accurate data points this polynomial can be constructed to interpolate these *n* points by, for example, Lagrange polynomial or Newton divided difference algorithms. But, for large *n*, it is also well known that polynomials of high degree can produce unwanted oscillations between the interpolated points. It is crucial then to approximate sets of data with as low degree polynomials as possible. Of course these polynomials may or may not interpolate the data points but may be made to come as close to them as possible. The ability to create highly flexible approximants from low-degree polynomials is a significant advantage of functions called splines.

Given a set of $\hat{r}$ real numbers, satisfying $a<\xi_{1}<\xi_{2}<\cdots <\xi_{\hat{r}}<b$, a spline function, *F*(*x*), of order *p* (or degree *p−*1) with knots $\xi_{1},\;\xi_{2},\cdots ,\;\xi_{\hat{r}}$ is a function that satisfies two properties: (1) in each of the intervals *x* ≤ ξ_1_; ξ*_j_*_−1_ ≤ *x* ≤ ξ*_j_*, $\left(j=2,3,\cdots ,\hat{r}\right)$; $\xi_{\hat{r}}\leq x$, *F*(*x*) is a polynomial of degree *p*−1 or less. (2) *F* (*x*) and its derivatives of orders 1,2, ⋯,*p*−2 are continuous. This would mean, for example, a spline function of order four would be constructed from polynomials of degree three (cubic) or less on the intervals *x* ≤ ξ_1_; ξ*_j_*_−1_ ≤ *x* ≤ ξ*_j_*, $\left(j=2,3,\cdots ,\hat{r}\right)$; $\xi_{\hat{r}}\leq x$ with continuous derivatives of orders one and two.

**Fig. 7:**
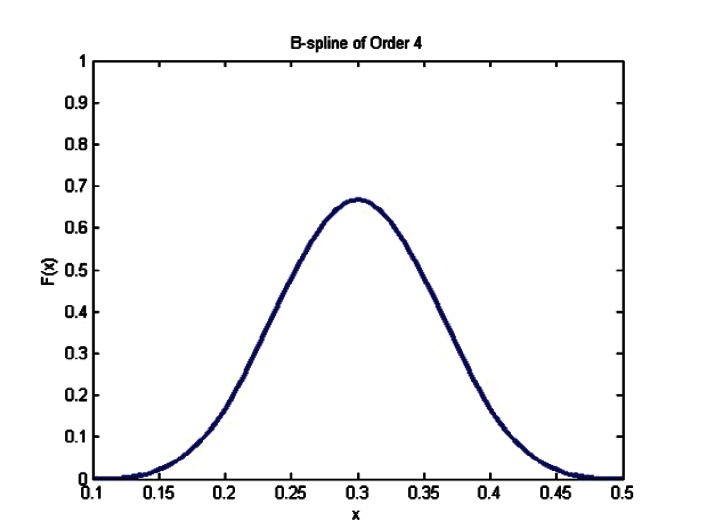
B-spline of Order 4 with knots at x = 0.1, 0.2, 0.3, 0.4, 0.5.

A well known mathematical technique to construct complicated functions is to form linear combinations of simpler functions, called basis functions. In the current paper, data sets will be approximated by linear combinations of special spline functions, called B-splines, for Basis splines. It is known that any spline function can be represented in terms of B-splines. This particular basis has the advantage of leading to computational algorithms that are elegant, efficient, and stable. Only B-splines of order four will be considered here. They are cubic splines that are non-zero only over four adjacent intervals between knots (see Fig. 7). The notation for a B-spline is *N_p, i_* (*x*), where *N_p, i_* (*x*), is zero everywhere except in the range *ξ_i–p_* <*x < ξ_i_*, where in this work *p* = 4. To simplify notation let *N_i_*(*x*) = *N*_4_, *_i_* (*x*). Then a B-spline of order four, or cubic B-spline, is a cubic spline with knots *ξ_i_*_−4_, *ξ_i_*_−3_, *ξ_i_*_−2_, *ξ_i_*_−1_, *ξ_i_* that is zero everywhere except in the range *ξ_i_*_−4_ <*x* < ξ_i_. N_i_(*x*) is defined uniquely except for a scaling factor and is conventionally taken to be positive throughout the range *ξ_i_*_−4_ < *x* < *ξ_i_* and has a single maximum value. Since *N_i_* (*x*) is a cubic spline it has continuous derivatives of order one and two at *ξ_i_*_−4_ and *ξ_i_*. These derivatives are zero at the endpoint knots.

To define a complete set of B-splines on the set of points $a<\xi_{1}<\xi_{2}<\cdots <\xi_{\hat{r}}<b$ it is necessary to introduce eight additional points at the boundaries given by *ξ*_−3_, *ξ*_−2_, *ξ*_−1_, *ξ*_0_, $\xi_{\hat{r}+1}$, $\xi_{\hat{r}+2}$, $\xi_{\hat{r}+3}$, $\xi_{\hat{r}+4}$. It is usual to have *ξ*_0_ = *a*, $\xi_{\hat{r}+1}=b$. With this augmented set of knots one can define $\hat{r}+4$ fundamental cubic B-splines, *N_i_*(*x*), $i=1,2,\cdots ,\hat{r}+4$. Then the general cubic B-spline has a unique representation in the range *a* ≤ *x* ≤ *b* of the form

(17) $$F(x)=\sum_{i=1}^{r}c_{i}N_{i}(x),$$

where $r=\hat{r}+4$.

There are computational advantages in using cubic B-splines. For any given *x*, all but four adjacent *N_i_*(*x*) are zero. In particular, if *x ∈* [*ξ_i_*_−1_, *ξ_i_*], the four nonzero cubic B-splines are *N_i_*(*x*), *N_i_*_+1_ (*x*), *N_i_*_+2_(*x*), *N_i_*_+3_ (*x*).

A least squares curve fitting problem to the data *a<x*_1_<*x*_2_<*⋯ <x_n_<b, y_q_* = *f*(*x_q_*), *q* = 1,⋯,*n*, becomes one of determining the coefficients *c_i_* as the least squares solution to the equations

(18) $$\sum_{i=1}^{r}c_{i}N_{i}(x_{q})\approx y_{q},\;q=1,2,\cdots ,n.$$

These may be written in matrix notation as

(19) Ac≈y,

where *A* is the *n × r* matrix whose element in column *i* of row *q* is *N_i_* (*x_q_*) and *c*, *y* are column vectors with elements *c_i_*, *y_q_*, *q* = 1,⋯, *n*, respectively. If the data points are arranged in increasing order of *x*, then the matrix *A* becomes a banded matrix with bandwidth four. For a more thorough discussion of B-splines and their computation see de Boor [11] and Cox [12].

There are two functions in the MATLAB Spline Toolbox that implement the evaluation of B-splines. A set of knots can be augmented at the ends by the function **augknt** and the B-splines and their derivatives can be evaluated by the function **spcol**.

#### 4.2.2 Tensor Products of Cubic B-Splines in Two Variables

In this section the B-spline concept will be extended to two dimensions in order to fit two dimensional scattered data by a surface function. In this surface-fitting problem data points (*x_q_*, *y_q_*), *q* = 1,2,⋯,*n*, and values at these points, *z_q_* = *f*(*x_q_*, *y_q_*), *q* = 1,2,⋯,*n*, are given. The surface model used to fit these data points will involve sums of products of B-splines.

To introduce this model, define a rectangle, say *R*, by *a* ≤ *x* ≤ *b*, *c* ≤ *y* ≤ *d* in the (*x*, *y*) plane. The definition does not restrict the data points to the Euclidean plane. They could, for example be angular coordinates, as will be seen later. The rectangle is subdivided by sets of knots $\xi_{i},\;i=1,2,\cdots ,\hat{r}$ and *η_j_*, $j=1,2,\cdots ,\hat{s}$, where $a<\xi_{1}<\xi_{2}<\cdots <\xi_{\hat{r}}<b$ and $c<\eta_{1}<\eta_{2}<\cdots <\eta_{\hat{s}}<d$, where $\hat{r}$, $\hat{s}$ are indices, not necessarily equal and *a*, *b*, *c*, *d* are the bounds on the rectangle *R*. The knots are then extended by eight in each dimension as done in the one dimensional case. These knots divide the rectangle *R* into rectangular panels in the plane given by *R_ij_*, $i=1,2,\cdots ,\hat{r}$, $j=1,2,\cdots ,\hat{s}$. Then, a basis set of splines for this pairing of knots can be constructed as products of B-splines *N_i_*(*x*)*N_j_*(*y*). In fact, the surface spline model is given by

(20) $$F(x,y)=\sum_{i=1}^{r}\sum_{j=1}^{s}c_{ij}N_{i}(x)N_{j}(y),$$

called a tensor product of splines, where $r=\hat{r}+4$ and $s=\hat{s}+4$.

As in the one dimensional case, these tensor product splines have a number of advantages. First of all, the basis functions *N_i_*(*x*)*N_j_*(*y*) are each non-zero over a rectangle composed of sixteen panels in a 4 × 4 arrangement. In particular *N_i_*(*x*)*N_j_*(*y*) is non-zero only when *ξ_i_*_−4_ ≤ *x* ≤ *ξ_i_* and *η_j_*_−4_ *≤ y ≤ η_j_*. Next, if (*x_q_*, *y_q_*), *q* = 1,2,⋯, *n* and values at these points, *z_q_* = *f*(*x_q_*, *y_q_*), *q* = 1,2,⋯,*n* are given, then the fitting problem can be formulated as finding the least squares solution of

(21) $$\sum_{i=1}^{r}\sum_{j=1}^{s}c_{ij}N_{i}(x_{q})N_{j}(y_{q})\approx z_{q},\;q=1,2,\cdots ,n.$$

Again, this can be written in a matrix form as

(22) Ac≈z,

where *A* is now a matrix with *n* rows and *rs* columns, and *z* is a column vector with *n* rows. The elements in row *q*, *q* = 1,2,⋯, *n*, *of A* are formed as follows. We start with a *fixed j*, *j* = 1,2,⋯, *s*, and let *i*, *i* = 1,2,⋯, *r* vary for that *j*. Then, the element in column (*j* −1)*r* + *i* for row *q* is given by *N_i_*(*x_q_*)*N_j_*(*y_q_*). The *j* is then incremented and the *i*’s varied again. The end resulting column values in *A* for row *q* would look like *N*_1_(*x_q_*)*N*_1_(*y_q_*) *N*_2_(*x_q_*)*N*_1_(*y_q_*) ⋯ *N_r_*(*x_q_*)*N*_1_(*y_q_*) *N*_1_(*x_q_*)*N*_2_(*y_q_*) ⋯ *N_r_*(*x_q_*)*N*_2_(*y_q_*) ⋯ *N*_1_(*x_q_*)*N_s_*(*y_q_*) ⋯ *N_r_*(*x_q_*)*N_s_*(*y_q_*). For a full discussion of tensor products of B-splines see de Boor [11], Eberly [13], and Rogers and Adams [14].

#### 4.2.3 An Issue in Computing Tensor Product B-Splines and the Relation to Least Squares

Unfortunately, for some choices of knots the resulting matrix *A* in (22) might be rank deficient and it would not be possible to use the normal equations to solve the least squares problem. This can potentially happen in the case of widely scattered data, where some of the knot panels do not contain data points. This problem can be solved, though, by using the matrix singular value decomposition.

Assume that a tensor product spline has been computed as described in Sec. 4.2.2. A least squares fit can be done to the scattered data as follows. Since the matrix *A* could be rank deficient, with potentially zero rows or columns, we cannot rely on the standard normal equations for determining the coefficients, but using the matrix singular value decomposition provides a convenient substitute (see Golub and Van Loan [15]). In the singular value decomposition of the matrix *A*, with the number of rows larger than the number of columns, the matrix is decomposed into the product of a column-orthogonal matrix *U*, a diagonal matrix *S*, with diagonal elements *S_i_*, and the transpose *V*′ of a square orthogonal matrix, so that *A* = *USV*′. In order to solve the problem *Ac* = *z* we compute *c* = *V*(*S*^+^(*U*′ *z*)), where

(23) $$S_{i}^{+}=\{\begin{array}{ll} \frac{1}{S_{i}} & S_{i}\neq 0\\ 0 & \text{otherwise} \end{array}$$

defines the generalized inverse of *S* and *U*′ is the transpose of *U*. A tolerance is used to determine which of the small singular values in the decomposition should be considered zero.

### 4.3 A Knot Selection Algorithm

In a least squares data fitting process involving B-spline basis functions, the resulting model residuals are sensitive to the knot placement for the B-splines. The selection of B-spline knots in order to achieve as small a residual as possible during the least squares process is a nontrivial task. One would be extremely lucky to manually select a set of knots that could achieve a very small least squares residual. In this section we will describe a heuristic algorithm that, in practice, generates a set of knots that produces small least squares residuals. The strategy involves an iterative knot insertion algorithm. An initial set of knots is selected by the algorithm user and, at each iteration of the algorithm, new knots are inserted in the vicinity of the previous fit where the local residuals are the largest. The knots are not moved once they have been inserted. The iterative algorithm has a stopping criteria based on a statistical test.

First we will discuss the knot insertion algorithm. It is based on a strategy suggested by Dierckx [3]. At the beginning of each iteration the assumption is that there exists a current set of knots. In the first iteration of the algorithm these would be an initial set chosen by the user. The tensor product of the B-splines is computed at the data points, a least squares fit is made to the data, and the current absolute residuals of the fit are computed at each data point. The current knots divide the (*x*, *y*) plane into rectangles. Some rectangles have data points and others don’t. Let the knots at the *k*-th iteration be labeled $a<\xi_{1}^{\left(k\right)}<\xi_{2}^{\left(k\right)}<\cdots <\xi_{r_{k}}^{\left(k\right)}<b$ along the *x*-axis and $c<\eta_{1}^{\left(k\right)}<\eta_{2}^{\left(k\right)}<\cdots <\eta_{s_{k}}^{\left(k\right)}<d$ along the *y*-axis. The (*k* + 1)-iteration begins by associating all of the data points with the knot panels in which they fall. Let the index pair *ij* indicate the *R_ij_* -th panel defined by *ξ_i_* ≤ *x* ≤ *ξ_i_*_+1_, *η_j_ ≤ y ≤ η_j+_*_1_. Then suppose the data values $\left(x_{1}^{\left(ij\right)},y_{1}^{\left(ij\right)}),\cdots ,(x_{ri_{j}}^{\left(ij\right)},y_{r_{\text{ij}}}^{\left(ij\right)}\right)$, fall into the *R_ij_-th* panel. Let *F_k_* (*x*, *y*) be the least squares B-spline function fit to the data at the *k-th* iteration. We next compute the residuals of the fit at all of the data points

(24) $${\text{Resid}}_{kd}=z_{q}-F_{k}(x_{q},y_{q}),\;q=1,\cdots ,n.$$

From these residuals we form the sums of squares of the residuals that fall within the knot intervals. The sums are separated into the *x* direction and the *y* direction as follows.

(25) $$\begin{array}{l} \delta_{i,x}^{\left(k\right)}=\sum_{q}\left\{{\text{Resid}}_{kq}^{2}:\xi_{i}^{\left(k\right)}\leq x_{q}<\xi_{i+1}^{\left(k\right)}\right\},i=1,2,\cdots ,r_{k},\\ \delta_{j,y}^{\left(k\right)}=\sum_{q}\left\{{\text{Resid}}_{kq}^{2}:\eta_{j}^{\left(k\right)}\leq y_{q}<\eta_{j+1}^{\left(k\right)}\right\},\;j=1,2,\cdots ,s_{k}. \end{array}$$

What is meant here, for example in the case of $\delta_{i,x}^{\left(k\right)}$, is that the sum, at the *k*-th iteration of Resid ${}_{kq}^{2}$, is taken over all pairs of points (*x_q_*, *y_q_*) such that $\xi_{i}^{\left(k\right)}\leq x_{q}<\xi_{i+1}^{\left(k\right)}$. This is done for each *i* = 1,2, ⋯, *r_k_*.

We next find the maximums of these sums of squared residuals.

(26) $$\begin{array}{l} \delta_{u,x}^{\left(k\right)}=\max\left\{\delta_{i,x}^{\left(k\right)}:i=1,2,\cdots ,r_{k}\right\},\\ \delta_{\upsilon ,y}^{\left(k\right)}=\max\left\{\delta_{j,y}^{\left(k\right)}:j=1,2,\cdots ,s_{k}\right\}, \end{array}$$

where *u = i* for some *i* = 1,2,⋯, *r_k_* and υ = *j* for some *j* = 1,2,⋯, *s_k_*. The next step is to add one knot at a time at each iteration. In particular, a knot is added in the *x* direction, if $\delta_{u,x}^{\left(k\right)}>\delta_{\upsilon ,y}^{\left(k\right)}$ or added in the *y* direction, if $\delta_{u,x}^{\left(k\right)}<\delta_{\upsilon ,y}^{\left(k\right)}$. The knot added is positioned based upon a weighted average of *x* or *y* data points in the columns or rows determined by the knot intervals with the maximum residual errors determined by (26). In particular, the position are given by

(27) $$\begin{array}{l} \xi^{\left(k+1\right)}=\sum_{q}\left\{\frac{{\text{Resid}}_{kq}^{2}}{\delta_{u,x}^{\left(k\right)}}x_{q}:\xi_{u}^{\left(k\right)}\leq x_{q}<\xi_{u+1}^{\left(k\right)}\right\},\\ \eta^{\left(k+1\right)}=\sum_{q}\left\{\frac{{\text{Resid}}_{kq}^{2}}{\delta_{\upsilon ,y}^{\left(k\right)}}y_{q}:\eta_{\upsilon }^{\left(k\right)}\leq y_{q}<\eta_{\upsilon +1}^{\left(k\right)}\right\}. \end{array}$$

We note that

(28) $$\begin{array}{l} \sum_{q}\left\{\frac{{\text{Resid}}_{kq}^{2}}{\delta_{u,x}^{\left(k\right)}}x:\xi_{u}^{\left(k\right)}\leq x_{q}<\xi_{u+1}^{\left(k\right)}\right\}=1,\\ \sum_{q}\left\{\frac{{\text{Resid}}_{kq}^{2}}{\delta_{\upsilon ,y}^{\left(k\right)}}:\eta_{\upsilon }^{\left(k\right)}\leq y_{q}<\eta_{\upsilon +1}^{\left(k\right)}\right\}=1, \end{array}$$

so that (27) represents weighted averages of all of the data pairs (*x_q_*, *y_q_*) satisfying $\xi_{u}^{\left(k\right)}\leq x_{q}<\xi_{u+1}^{\left(k\right)}$ and $\eta_{\upsilon }^{\left(k\right)}\leq y_{q}<\eta_{\upsilon +1}^{\left(k\right)}$.

The knots are then reindexed as necessary and *F_k_*_+1_(*x*,*y*) is computed as the least squares B-spline function fit to the data at the (*k* + 1)-iteration based on the new knot set. The iterations continue until the stopping criteria is met.

The stopping criteria for the *k*-th iteration used in this algorithm is based on the use of the *R*^2^-statistic, called the coefficient of multiple determination (see Draper and Smith [8]). *R*^2^ is the square of the correlation between the vector of observed data, *z_q_*, *q* = 1,⋯, *n*, and the least squares predicted data, *F_k_*(*x_q_*, *y_q_*), *q* = 1,⋯, *n*. The statistic satisfies 0 ≤ *R*^2^ ≤ 1. This statistic is often used as a measure of how well the regression equation explains the variation in the data. It is known that in building models based on adding terms in the regression equation, care must be taken in using this statistic. However, in the current algorithm, the statistic is used in a somewhat non-conventional manner. We use it as a measure of the benefit of adding more knots to the tensor product spline model. The knot selection algorithm is terminated when *R*^2^ > 0.98.

Although the knot placement algorithm is heuristic, coupling it with the *R*^2^-statistic has shown good convergence in practice. It is reasonable to expect this, since knots are placed in intervals in which the fits at a previous iteration showed the largest local error. Placing a knot there allows extra flexibility for those areas.

### 4.4 Data Smoothing by Tikhonov Regularization

As noted in Sec. 4.2.3, the data distribution can lead to a rank deficient least squares matrix. Even though a fit can be computed using SVD, evaluations of the fitted function at some points can lead to unwanted oscillations. It is necessary to introduce a smoothing procedure by regularization. Regularization is a way of introducing extra information to the least squares objective function in order to control overfitting of the data. It is the overfitting of data that can lead to the unwanted oscillations. In the present work a penalty term is introduced to control the smoothness of the resulting fitted function. The objective function will then balance the data fitting with the smoothness of the fit. The second partial derivatives of the tensor product B-splines will be introduced as the smoothing operators. The objective function will take the form

(29) $$\begin{array}{l} O(c)={\sum_{q=1}^{n}\left[\sum_{i=1}^{r}\sum_{j=1}^{s}c_{ij}N_{i}(x_{q})N_{j}(y_{q})-z_{q}\right]}^{2}\\ \;+{\sum_{p=1}^{t}\left[\sum_{i=1}^{r}\sum_{j=1}^{s}\lambda c_{ij}N_{i}^{\prime \prime }(u_{p})N_{j}(\upsilon_{p})\right]}^{2}\\ \;+{\sum_{p=1}^{t}\left[\sum_{i=1}^{r}\sum_{j=1}^{s}\lambda c_{ij}N_{i}(u_{p})N_{j}^{\prime \prime }(\upsilon_{p})\right]}^{2}. \end{array}$$

*λ* is called a Tikhonov parameter.

In the objective function the data values are given by (*x_q_*, *y_q_*, *z_q_*), *q* = 1,⋯,*n*. The smoothing terms will be evaluated at a new set of points chosen so that every knot panel has an equal number of points assigned to the panel. In the current case there will be a total of *t* points throughout the knot panels given by (*u_p_*,*υ_p_*), *p* = 1, ⋯, *t*.

To write this in matrix form we will define two new matrices *B*_1_ and *B*_2_ as follows. For *m* = 1,2,⋯, *rs* let (*i_m_*, *j_m_*) be such that *m* = (*j_m_* −1) r + *i_m_*. Then define the matrix elements

(30) $$\begin{array}{l} A(q,m)=N_{i_{m}}(x_{q})\;N_{j_{m}}(y_{q})\\ B_{1}(p,m)=N_{i_{m}}^{"}(u_{p})\;N_{j_{m}}(\upsilon_{p})\\ B_{2}(p,m)=N_{i_{m}}(u_{p})\;N_{j_{m}}^{"}(\upsilon_{p}). \end{array}$$

We can rewrite (29) in the form

(31) $$\begin{array}{c} O(c)={\sum_{q=1}^{n}\left[\sum_{m=1}^{rs}c_{m}A(q,m)-z_{q}\right]}^{2}\\ \;+{\sum_{p=1}^{t}\left[\lambda \sum_{m=1}^{rs}c_{m}B_{1}(p,m)\right]}^{2}\\ \;+{\sum_{p=1}^{t}\left[\lambda \sum_{m=1}^{rs}c_{m}B_{2}(p,m)\right]}^{2}\\ \;={\left\|Ac-z\right\|}^{2}+{\left\|\lambda B_{1}c\right\|}^{2}+{\left\|\lambda B_{2}c\right\|}^{2}\\ ={\left\|\left(\begin{array}{c} A\\ \lambda B_{1}\\ \lambda B_{2} \end{array}\right)c-\left(\begin{array}{c} z\\ 0\\ 0 \end{array}\right)\right\|}^{2}. \end{array}$$

The matrix dimensions in this objective function are:

$$A\;\mathrm{is}\;n\times rs,B_{1}\;\text{is}\;t\times rs,B_{2}\;\text{is}\;t\times rs,\left(\begin{array}{c} A\\ \lambda B_{1}\\ \lambda B_{2} \end{array}\right)$$

$$\text{is}\;(n+2t)\times rs,\text{and}\;(\begin{array}{c} z\\ 0\\ 0 \end{array})\;\text{is}\;(n+2\;t)\times 1.$$

If we perform a *QR* decomposition, then

(32) $$\left(\begin{array}{c} A\\ \lambda B_{1}\\ \lambda B_{2} \end{array}\right)=QR,$$

where *Q* is (*n* + 2*t*) × (*n* + 2*t*) and *R* is (*n* + 2*t*) × *rs. Q* is orthogonal, so that *Q^T^ Q* = *I*, and *R* is zero except in its upper right corner. Let $\hat{R}$ be the *rs × rs* upper triangular portion of *R* and let

(33) $$d=Q^{T}\left(\begin{array}{c} z\\ 0\\ 0 \end{array}\right).$$

Then, let $\hat{d}$ be the upper *rs* entries of *d*. The minimum of *O*(*c*) then satisfies $\hat{R}c=\hat{d}$. For a more complete discussion of the *QR* method see Golub and Van Loan [15].

At this point we need to discuss the selection of the Tikhonov parameter, λ. A number of methods for choosing the parameter have been discussed in the literature (see [16, 17, 18, 19, 20]). For this work, though, we used a graphical method that led to the selection of a parameter in a few iterations. Before continuing to discuss the selection of the Tikhonov parameter for the current study we need to change the coordinates of the original probe points to spherical coordinates defined on a rectangle.

#### 4.4.1 A Coordinate Transformation

Since our tensor product B-spline requires a rectangular grid, we convert our CMM data to spherical coordinates. We start with a given set of *n* points, (*x_i_*, *y_i_*, *z_i_*), *i* = 1, 2, ⋯,*n*, for some *n*, on a surface. As measured, these points are given relative to the CMM origin. The first step is to center the data by using the center-of-data mass of the data points. This establishes an origin at the center-of-data mass point. It is done in order to establish a common reference point interior to the measured data points. It also simplifies writing vectors from the origin to the data points and allows the introduction of spherical coordinates. The center-of-data point is given by

(34) $$\begin{array}{l} \bar{x}=\frac{1}{n}\sum_{i=1}^{n}x_{i}\\ \bar{y}=\frac{1}{n}\sum_{i=1}^{n}y_{i}\\ \bar{z}=\frac{1}{n}\sum_{i=1}^{n}z_{i}. \end{array}$$

Since the data set is enclosed in a near-spherical bounded region, it is reasonable to identify the Euclidean data points with spherical coordinates. This will map the points on the sphere to a rectangular surface where the surface coordinates are designated by *θ*, *ϕ* and the height of a surface point is given by *R*(*ϕ*, *θ*). In order to use spherical coordinates to represent points on the boundary of a surface, we need to restrict our analysis to surfaces that are called star-shaped. These are surfaces in which a ray drawn from the center-of-data mass intersects the boundary in a unique point. Whereas, in the definition of the B-splines, we used the coordinates (*x,y*) we will now use (*ϕ*, *θ*) and build B-splines in terms of these spherical coordinates. Euclidean coordinates will now refer to the measured data points. This switch in notation should not, it is hoped, cause too much confusion.

**Fig. 8:**
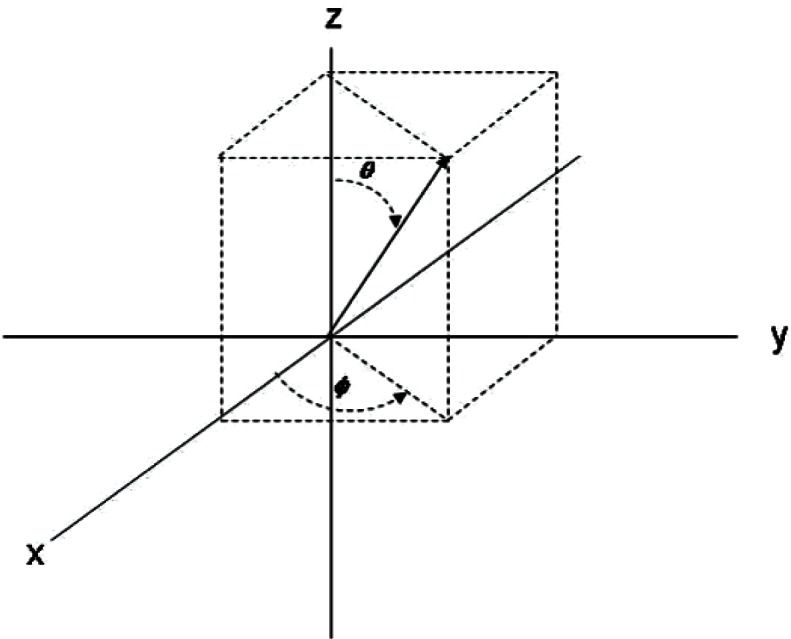
Spherical Coordinate angles. *θ* is the colatitude and *ϕ* is the azimuth.

To each data point there is a vector from $\left(\bar{x},\bar{y},\bar{z}\right)$ to (*x_i_*, *y_i_*, *z_i_*), given by $V_{i}=(x_{i},y_{i},z_{i})-(\bar{x},\bar{y},\bar{z})$. Furthermore, any point within the bounding sphere can be identified by spherical coordinates of the form *S*(*R*,*ϕ*, *θ*) = (*R* sin(*θ*)cos(*ϕ*), *R* sin(*θ*)sin(*ϕ*), *R* cos(*θ*)), where *x = R* sin(*θ*)cos(*ϕ*), *y = R* sin(*θ*)sin(*ϕ*), *z* = *R* cos(*θ*), for 0 ≤ *θ* ≤ π, 0 ≤ *ϕ* ≤ 2π. *θ is* referred to as the colatitude and *ϕ* is referred to as the azimuth (Fig. 8). Table 2 associates the three dimensional octants with their spherical coordinates (where we have suppressed *R*). Therefore, the Euclidean coordinates were converted to *θ*, *ϕ* angles on a rectangle [0, π] × [0, 2π]. The height, *R* (*ϕ*, *θ*), at each *θ*, *ϕ* is taken as the estimated radius from the center-of-data mass of all of the Euclidean coordinates.

**Fig. 9:**
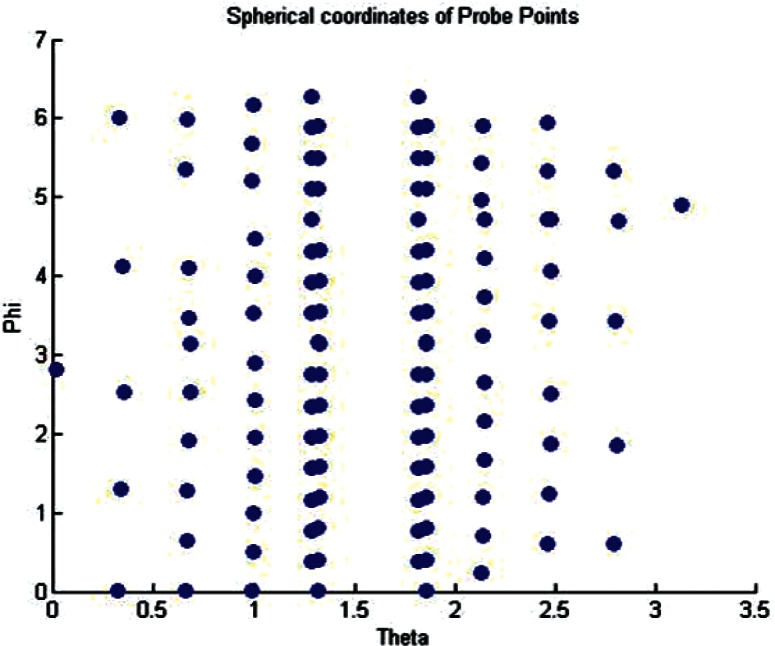
Plot of the *θ*, *ϕ* Coordinates of the Probe Points for the Coarse Data.

As an illustration, the results of the conversion of the coarse probe points for the Green phantom from Euclidean coordinates to *ϕ*, *θ* coordinates are shown in Fig. 9. We note the density of data points near the equator is higher than towards the poles at *θ* = 0 and *θ* = π. Unfortunately the distribution of data points was dictated by the software controlling the CMM. The lack of data points near the poles leads to a well known problem, called the *Pole Problem* in the literature (see Dierckx [3]). It creates rank deficient matrices during the least squares fitting process. Section 4.2.3 discussed how the singular value decomposition can be used to handle this problem.

#### 4.4.2 Choosing a Tikhonov Parameter

Once the original probe data had been converted to spherical coordinates, the iterative selection of the Tikhonov parameter for the current volume estimation problem proceeded as follows. First, a set of knots was selected with *λ* = 0 (i.e., no regularization terms) in order to produce *an R*^2^ > 0.98 as described in Sec. 4.3. We found that, for all of the data sets examined, only a very few extra knots were added beyond the initial set used to begin the knot selection process. This led to a rapid convergence to a set of final knots in all cases. These knots formed panels in the rectangle [0, π] *×* [0, 2π]. Next, nine points were uniformly chosen in each panel and the regularization terms formed. Numerical experimentation showed that the use of nine points in each panel provided sufficient extra data to the regularization terms in order to smooth the final fits. An initial Tikhonov parameter, *λ*, was chosen and the objective function (29) was minimized by the *QR* method discussed above. An initial grid of 40 *θ* and 80 *ϕ* values was generated in the rectangle [0,π] × [0, 2π]. This grid was finally fixed on for selecting a Tikhonov parameter since further numerical experimentation showed that the response surface of the radii for denser grids did not change the final range of the radii. The coefficients, *c*, computed to minimize the objective function (29), were then inserted into the linear form *Ac*, where *A* was the tensor product matrix formed from all of the 40 × 80 grid points. The end resulting radii at the grid values were then computed and the spherical volumes for those radii were computed. The maximum and minimum sphere volumes over the entire 40 × 80 grid were determined. To select the appropriate Tikhonov parameter, *λ* values over a range were used and the differences between the maximum and minimum volumes were plotted. The value of *λ* that produced the minimum difference was selected as the working *λ*. For the current volume estimation problem, the final *λ* was *λ* = 0.32.

**Fig. 10:**
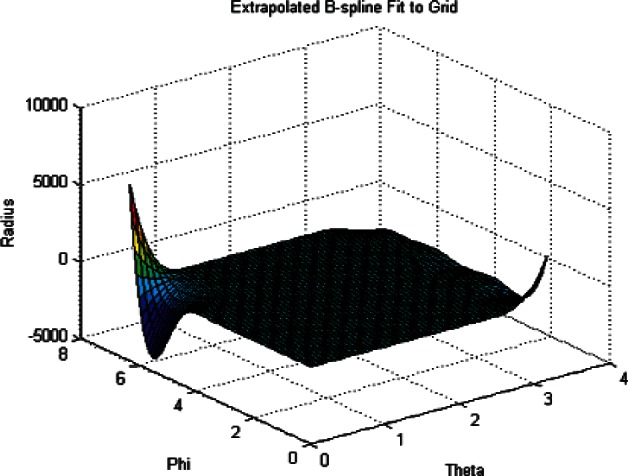
Plot of Radius Data on a 40 by 80 Grid. *λ* = 0.

To illustrate the effect of using regularization terms to smooth the least squares fit of the radii see Fig. 10.

**Fig. 11:**
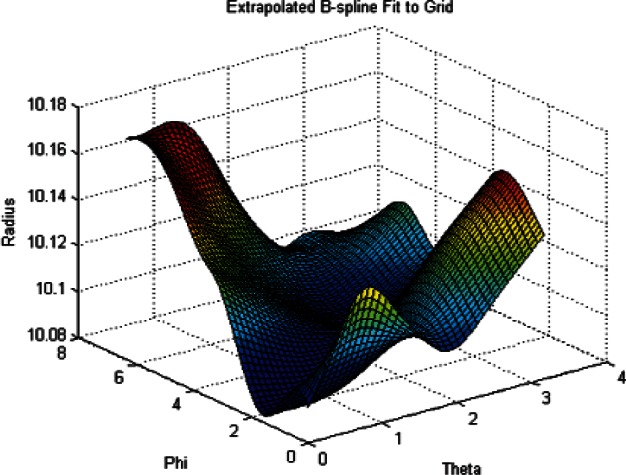
Plot of Radius Data on a 40 by 80 Grid. *λ* = 0.32.

This figure shows the radii data for the case of *λ* = 0, or no regularization. Note the large radii oscillations at the boundaries (a range of approximately 10,000 mm). Now see Fig. 11 and note the narrow range (approximately 0.1 mm) of the radii over the entire grid. This clearly shows the effect of the regularization terms on the least squares fit. The data used for these plots was the coarse Green phantom data.

## 5. Volume Estimation by Surface Triangulation

We could now estimate the volume using the triangulation method described in Sec. 2 by dividing the phantom surfaces into triangular surface patches as follows. First of all we partitioned the phantom surfaces at grid points located at the colatitude angles *θ*_1_ = 0 <*θ*_2_ < ⋯<*θ*_υ_<*θ*_υ_*_+_*_1_ = π from the north pole to the south pole and azimuthal angles *ϕ*_1_ = 0 <*ϕ*_2_ < ⋯ <*ϕ_h_< ϕ_h_*_+1_ = 2π around the phantom surface, where υ stands for vertical and *h* for horizontal. Since these grid points did not necessarily fall at the measured data points, a radius, *R*(*ϕ θ*), was calculated at the grid points from the regularized fitted B-spline surface model. At the north and south poles the radius was taken as the median value, *R_north_*, of the grid point values *R*(*ϕ* 0), *i* = 1,⋯, *h*, for the north pole and *R_south_*, the median value of *R*(*ϕ_i_*,π), *i* = 1,⋯, *h*,. The spherical coordinates of all of the grid points were converted to Euclidean coordinates on the surface by

(35) $$\begin{array}{l} x=R\;\sin(\theta )\;\cos\;(\phi )\;0\leq \theta \leq \pi ,\\ y=R\;\sin(\theta )\sin(\phi )\;0\leq \phi \leq 2\pi ,\\ z=R\;\cos(\theta ). \end{array}$$

At this point we could apply the Divergence Theorem method of Sec. 2. The patches at the north poles were easily constructed to be triangular as part of the process of determining the contribution of each patch to the phantom volumes. In particular, at the north pole the designated point was (*x*_1_, *y*_1_, *z*_1_) = (0, 0, *R_north_*). We then iterated through the spherical coordinate points (*ϕ_i_*, *θ*_2_), *i* = 1,⋯, *h*. At each of these angle pairs there was a value *R*(*ϕ_i_*, *θ*_2_), *i* = 1,⋯, *h*. We generated the volume by adding up the contributions of each patch to the volume total. We did this by initializing a variable, vol, for the volume, to zero. We then started to generate the contribution from the first layer of patches at the north pole. As noted above, we set the north pole to (*x*_1_, *y*_1_, *z*_1_) and then set

$$\begin{array}{l} x_{2}=R(\phi_{1},\theta_{2})\sin(\theta_{2})\cos(\phi_{1}),\\ y_{2}=R(\phi_{1},\theta_{2})\sin(\theta_{2})\sin(\phi_{1}),\\ z_{2}=R(\phi_{1},\theta_{2})\cos(\theta_{2}), \end{array}$$

and

$$\begin{array}{l} x_{3}=R(\phi_{2},\theta_{2})\sin(\theta_{2})\cos(\phi_{2}),\\ y_{3}=R(\phi_{2},\theta_{2})\sin(\theta_{2})\sin(\phi_{2}),\\ z_{3}=R(\phi_{2},\theta_{2})\cos(\theta_{2}). \end{array}$$

These were set in a positive orientation.

**Fig. 12:**
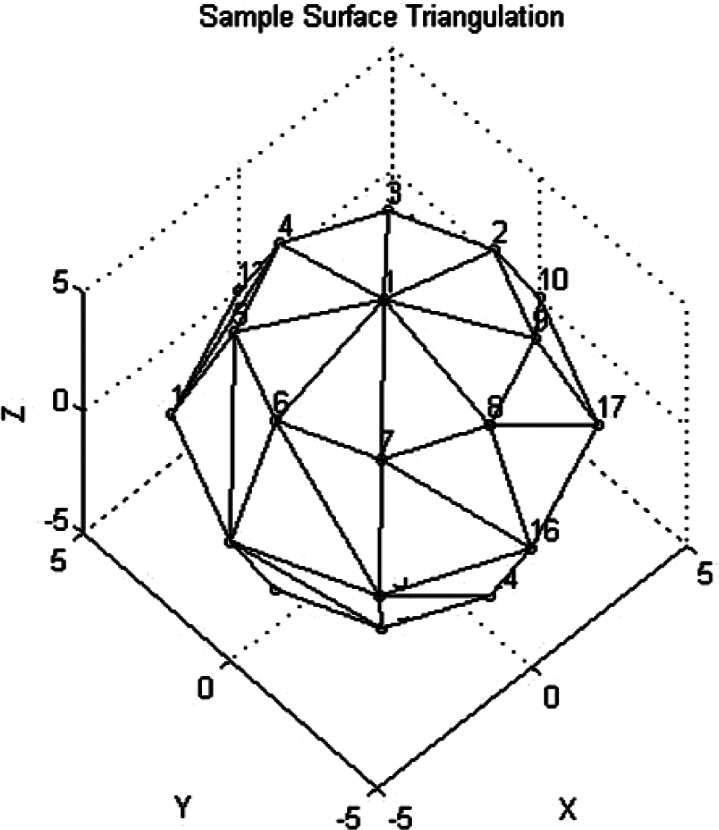
A Sample Triangulation of a Sphere Viewed from the North Pole (Vertex 1).

In order to make this process more concrete, we have included a sample surface triangulation with υ = 5 colatitude angles and *h* = 8 azimuthal angles in Fig. 12. In this figure the Euclidean grid points have been indexed. The North Pole (*x*_1_, *y*_1_, *z*_1_) is designated by the index 1. In the first step described above the points (*x*_2_, *y*_2_, *z*_2_) and (*x*_3_, *y*_3_, *z*_3_) are indexed in Fig. 12 by points 2 and 3 respectively. Next we computed the outward normal to the triangle patch by forming the vectors υ_1_ = (*x*_2_, *y*_2_, *z*_2_) −(*x*_1_, *y*_1_, *z*_1_), υ_2_ = (*x*_3_, *y*_3_, *z*_3_) −(*x*_1_, *y*_1_, *z*_1_), for triangle 123 in Fig. 12 and then formed the normalized cross product

(36) $$\hat{n}=\frac{\upsilon_{1}\times \upsilon_{2}}{\left\|\upsilon_{1}\times \upsilon_{2}\right\|}.$$

We then computed the contribution that this patch made to the volume as

(37) $$\begin{array}{l} \text{vol}=\text{vol}+(\hat{n}\cdot (x_{1},y_{1},z_{1}))\\ \;\left\{\hat{n}\cdot \left(\sum_{j=1}^{3}\left(y_{j}z_{j+1-k}-y_{j+1-k}z_{j}),(z_{j}x_{j+1-k}-z_{j+1-k}x_{j}\right),(x_{j}y_{j+1-k}-x_{j+1-k}y_{j})\right)\right\}, \end{array}$$

where

(38) $$k=\{\begin{array}{cc} 0 & 1\leq j<3\\ 3 & j=3 \end{array}$$

is set in order to compensate for the cyclical vertex indexing around the triangle patch. Formula (37) is a combination of Eq. (6), where $\left(\hat{n}\cdot (x_{1},y_{1},z_{1})\right)$ is the coefficient *c* in (6), and the second factor is a compact form of Eq. (11). The factors 1/3 from (6) and 1/2 from (11) were combined as a multiple of 1/6 after all of the summations had been performed.

We proceeded to the next patch in the North Pole layer. Again (*x*_1_, *y*_1_, *z*_1_) was the North Pole, indexed by 1 in Fig. 12, and we then used the previous computation to get *x*_2_ = *x*_3_, *y*_2_ = *y*_3_, *z*_2_ = *z*_3_ and set

$$\begin{array}{l} x_{3}=R(\phi_{3},\theta_{2})\sin(\theta_{2})\cos(\phi_{3}),\\ y_{3}=R(\phi_{3},\theta_{2})\sin(\theta_{2})\sin(\phi_{3}),\\ z_{3}=R(\phi_{3},\theta_{2})\cos(\theta_{2}). \end{array}$$

In Fig. 12 the new point (*x*_3_, *y*_3_, *z*_3_) is indexed by 4 in Fig. 12. The triangle of interest is now 134 in terms of indices. We computed the normalized cross product as for the first patch and then computed the contribution of the second patch to the volume using Eq. (37). We continued this process for *θ*_2_, *ϕ_i_*, *i* = 1,⋯, *h*. In Fig. 12 we would have proceeded with computing contributions to the volume by working through the indexed triangles 123, 134, 145, 156, 167, 178, 189, and 192.

We next computed the contributions of the middle layer patches in a two step process. We iterated through each *θ_j_*, *j* = 2,⋯, υ−1. The triangles at the South Pole were handled separately. For each *θ_j_*, *j = 2*,⋯, υ−1 and *ϕ_i_*, *i* = 1,⋯, *h*, the patches were defined first in terms of four vertices to create four sided patches. Each of these patches was then divided into two triangles. The four vertices of a rectangular patch were identified counterclockwise as (*θ_j_*, *ϕ_i_*, *R*(*i*, *j*)), (*θ_j+_*_1_, *ϕ_i_*, *R*(*i, j* + 1)), (*θ_j_*_+1_, *ϕ_i+_*_1_, *R*(*i* + 1, *j* + 1)), (*θ_j_*, *ϕ_i +_*_1_, *R*(*i* + *j*)). As an example, in Fig. 12 one of the patches is identified by indices, in counterclockwise order, as 8 16 17 9. These indices would be associated with vertices (*θ*_2_, *ϕ*_1_, *R*(1,2)), (*θ*_3_, *ϕ*_1_, *R*(1,3)), (*θ*_3_, *ϕ*_2_, *R*(2,3)), (*θ*_2_, *ϕ*_2_, *R*(2,2)). The four vertex patches were then divided into two triangles. For the first triangle in the rectangular patch we set

$$\begin{array}{l} x_{1}=R(i,\;j)\;\sin\;(\theta_{j})\;\cos\;(\phi_{i}),\\ y_{1}=R(i,\;j)\;\sin\;(\theta_{j})\;\cos\;(\phi_{i}),\\ z_{1}=R(i,\;j)\;\cos\;(\theta_{j}). \end{array}$$

$$\begin{array}{l} x_{2}=R(i,\;j+1)\;\sin\;(\theta_{j+1})\;\cos\;(\phi_{i}),\\ y_{2}=R(i,\;j+1)\;\sin\;(\theta_{j+1})\;\sin\;(\phi_{i}),\\ z_{2}=R(i,\;j+1)\;\cos\;(\theta_{j+1}). \end{array}$$

$$\begin{array}{l} x_{3}=R(i+1,\;j+1)\;\sin\;(\theta_{j+1})\;\cos\;(\phi_{i+1}),\\ y_{3}=R(i+1,\;j+1)\;\sin\;(\theta_{j+1})\;\sin\;(\phi_{i+1}),\\ z_{3}=R(i+1,\;j+1)\;\cos\;(\theta_{j+1}). \end{array}$$

This triangle in the example Fig. 12 would be 8 16 17. Again, the volume increment was computed as discussed previously. For the second triangle of the rectangular patch we maintained the same (*x*_1_, *y*_1_, *z*_1_) and set *x*_2_ = *x*_3_, *y*_2_ = *y*_3_, *z*_2_ = *z*_3_ and then set

$$\begin{array}{l} x_{3}=R(i+1,\;j)\;\sin\;(\theta_{j})\;\cos\;(\phi_{i+1}),\\ y_{3}=R(i+1,\;j)\;\sin\;(\theta_{j})\;\sin\;(\phi_{i+1}),\\ z_{3}=R(i+1,\;j)\;\cos\;(\theta_{j}). \end{array}$$

This triangle in the example Fig. 12 would be 8 17 9. Again the volume increment was computed as before. We continued the process for *θ_j_*, *j* = 2,⋯, υ−1 and and *ϕ_i_*, *i* = 1,⋯, *h.*

Finally, at the South Pole there were *h* triangles to include in the volume calculation. Their vertices were identified as follows

$$\begin{array}{l} x_{1}=R(i,\upsilon )\;\sin\;(\theta_{\upsilon })\;\cos\;(\phi_{i}),\\ y_{1}=R(i,\upsilon )\;\sin\;(\theta_{\upsilon })\;\sin\;(\phi_{i}),\\ z_{1}=R(i,\upsilon )\;\cos\;(\theta_{\upsilon }). \end{array}$$

$$\begin{array}{l} x_{2}=0,\\ y_{2}=0,\\ z_{2}=-R_{south}. \end{array}$$

$$\begin{array}{l} x_{3}=R(i+1,\upsilon )\;\sin\;(\theta_{\upsilon })\;\cos\;(\phi_{i+1}),\\ y_{3}=R(i+1,\upsilon )\;\sin\;(\theta_{\upsilon })\;\sin\;(\phi_{i+1}),\\ z_{3}=R(i+1,\upsilon )\;\cos\;(\theta_{\upsilon }). \end{array}$$

**Fig. 13:**
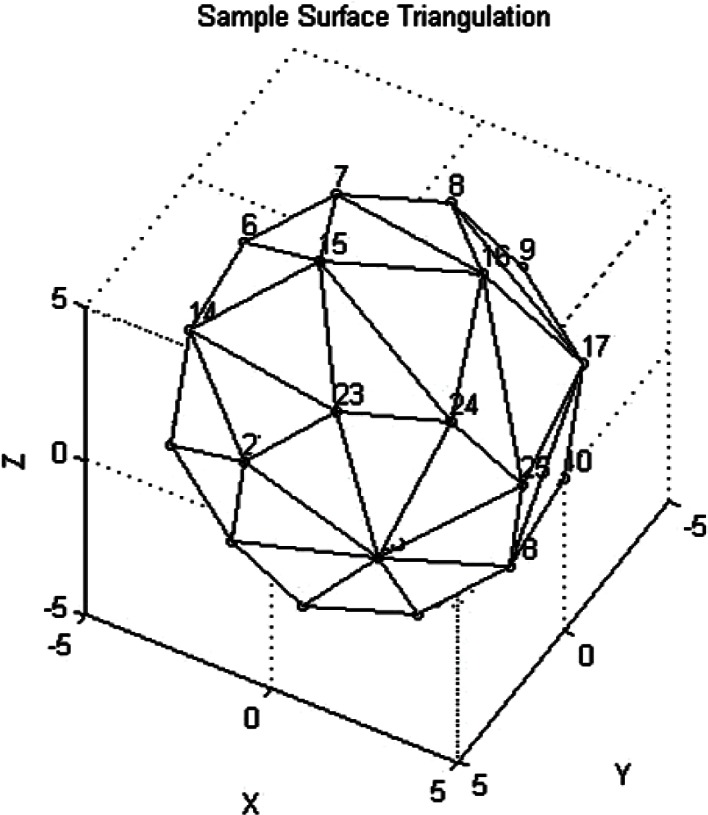
A Sample Triangulation of a Sphere Viewed from the South Pole (Vertex 26).

As an example, in Fig. 13 one of these triangles would be indexed by the points 23 26 24, where the South Pole is point number 26, although the South Pole index may be hard to see in the figure. The contribution of these triangles to the volume was computed as above. After all of the triangle contributions to the volume were computed, the final volume was taken to be vol = (1/6) vol. The factor 1/6 comes from the product of 1/3 from Sec. 2.1 and 1/2 from Sec. 2.2. The reader can refer back to these sections to see how the factors arose. The triangulation process can clearly be generalized to a denser surface triangulation.

## 6. Computational Results

Two computational processes were involved in generating the results for each object. The objects involved were the calibrated sphere and the two phantoms. First, sphere models were fit to an object. The second process involved three steps. The first step was to compute a good selection of knots for the tensor product spline function. The next step was to fit the tensor product spline function with the objective function modified by the regularization terms to the object. Finally, once the tensor spline function had been developed they were used to generate data at the vertices of the surface triangulation and to compute the volume using the Divergence Theorem.

### 6.1 Computational Results for the Spherical Model

Tables 3, 4, and 5 present the results obtained by fitting spherical models to the data from the calibrated sphere and the two phantoms. For the calibrated sphere there were five repeats for each of the coarse and dense data sets. Since the results of the point location repeats differed only at the micrometer level the data sets were combined by averaging to form two data sets representing the coarse and dense data sets for the calibrated sphere. Similarly, the point locations of the three position data sets for both the coarse and dense data sets for the phantoms differed only at the micrometer level. Therefore, by averaging of the three position data sets for coarse and dense data sets, two working data sets were formed for the Green and Pink phantoms. Since the output of the Nelder-Mead algorithm was a final polytope surrounding the minimizing parameter with the polytope, the final reported parameters were selected as the median value of the vertex values. These are the first four entries in the tables: Center x, Center y, Center z, and Radius. The units are millimeters. The fifth table entries are the spherical volumes based on the median radius value in cubic millimeters. The radius residuals were computed as the absolute value of

(39) $${\text{Resid}}_{i}=\sqrt{{\left(x_{i}-{\hat{c}}_{1}\right)}^{2}+{\left(y_{i}-{\hat{c}}_{2}\right)}^{2}+{\left(z_{i}-{\hat{c}}_{3}\right)}^{2}}-{\hat{c}}_{4}.$$

The sixth and seventh entries in the tables give the mean value and standard deviation of these residuals in millimeters. The eighth through the eleventh entries are the expanded uncertainties for the estimated Center x, Center y, Center z, and Radius, also in millimeters.

The results in these sections on spherical model fitting involved the non-linear model (39) fitting to the sparse data sets generated by the CMM. This process did not involve the B-spline algorithm. Therefore any differences between coarse and dense results were most likely the consequence of the point selection in the metrology of the artifacts.

#### 6.1.1 Results for the Spherical Model Fit to the Calibrated Sphere With Coarse and Dense Data

Table 3 gives the results of the fit of the sphere model to both the coarse (122 points) and the dense (181 points) data sets for the calibrated sphere. In both cases the average of the five repeat data sets was used for the fitting process. As can be seen, the two volume estimates differ by approximately 0.04 %. The radii estimates differ by about 0.01 %. There is a slightly larger difference when the spherical fit results are compared to the volume estimate based on the manufacturer estimated calibrated sphere diameter of 19.05 mm. This would lead to a volume estimate of 3619.8 mm^3^. Since the method of estimating the diameter by the manufacturer was proprietary we could not independently verify the measurement. We could only compare it against our sphere model fit results. The manufacturer measured radius differs from the computed radii in Table 3 by about 0.08 % whereas the volume estimate based on the measured radius differs from the volume estimates in Table 3 by about 0.2 %. If we expand the radii in Table 3 to a diameter we find the values 19.0651 millimeters in the coarse case and 19.0629 millimeters in the dense case. These differ by approximately 0.01 mm from the manufacture’s measured diameter. It would seem that these differences were sufficiently small so that the difference in value estimates for radius and volume were not considered significant. Although the estimated centers are slightly different, all other values are of the same order of magnitude. Based on these results we can accept that the CMM is producing accurate position data for spherical metallic artifacts. Tables 4 and 5, however, begin to show the consequences involved with attempting to measure slightly non-spherical and non-metallic artifacts with the CMM.

#### 6.1.2 Results for the Spherical Model Fit to the Phantoms With Coarse and Dense Data Sets

From Tables 4 and 5 it is clear that the Green phantom is more spherical than the Pink phantom as indicated by the residuals and the expanded uncertainties. This simply confirms the fact that the Pink phantom was more difficult to measure using the vacuum chuck due to its lack of sphericity. For example, the Mean Radial Residual for the Pink data is two orders of magnitude larger than for the Green data. The Standard Deviations for the Pink data are an order of magnitude greater, as are the Expanded Uncertainties. It is not clear how much the phantom material affected the results since it was difficult to set up a specific probe test for metallic versus phantom material. The artifacts would have to have been exactly the same size, positioned at exactly the right location, and probed at exactly the same coordinates to separate those factors from the material factor difference. There were no such comparable artifacts. We can make a guess, though, that there might be some effect due to probe force against the non-metallic material if we look at Table 3 and Table 4 for the Green phantom, the most spherical of the two phantoms. The Expanded Uncertainties for the sphere fit to the calibrated metallic sphere and the Expanded Uncertainties for the sphere fit to the Green phantom data differ by one to three orders of magnitude. Since the repeatability of the CMM measurements is at the 1 μm level, it is likely then that material difference had some significant affect on the difference in the uncertainties. It is a conjecture that this and the non-spherical shape of the Pink phantom account for a large part of the differences between the Pink phantom Expanded Uncertainties in Table 5, the Green phantom Expanded Uncertainties in Table 4, and the calibrated sphere Expanded Uncertainties in Table 3. For the Green phantom the radii estimates in Table 4 between the fits of the coarse and dense data sets is approximately 0.3 % and the volumes differ by approximately 0.8 %. Whereas, for the Pink phantom the radii estimates in Table 5 between the fits of the coarse and dense data sets are 0.4 % and the volumes differ by approximately 1.3 %. In both Tables 4 and 5 the expanded uncertainties for the dense data sets are approximately an order of magnitude better than the results for the coarse data sets. This suggests that the radius and volume estimates in the case of the dense data sets for both the Green and Pink phantoms are probably the more realistic values, at least in terms of a spherical model fit.

Table 1 shows that the diameter of the Green phantom is approximately 20 mm and that the diameter of the Pink phantom falls approximately between 20.0 mm and 18.4 mm. This would imply that the sphere model for the Green phantom should have a volume of about 4188.8 mm^3^. With the estimate of 0.3 mm uncertainty using the calipers, this would place the Green volume in the range of 4380.1 mm^3^ and 4003.1mm^3^. The estimated volumes in Table 4 are within this range for both the coarse and the dense data. The Pink phantom should have a volume between 4445.2 mm^3^ and 3104.8 mm^3^. Table 5 for the Pink phantom shows that for the coarse and dense data the volume estimates fall from about 3862 mm^3^ to about 3913 mm^3^. These volumes are within the expected range of the caliper measurements.

### 6.2 Computational Results for the B-Spline Model

In this section we will describe the method used to estimate volume uncertainties and discuss the results of using a B-spline surface model and the Divergence Theorem to estimate the phantom volumes. A nonpara-metric method to estimate the volume uncertainties was chosen since there did not exist any “ground truth” values for the Green and Pink FDA phantom volumes. All of the expanded uncertainty results are displayed in row two of Tables 6, 7, and 8.

**Fig. 14:**
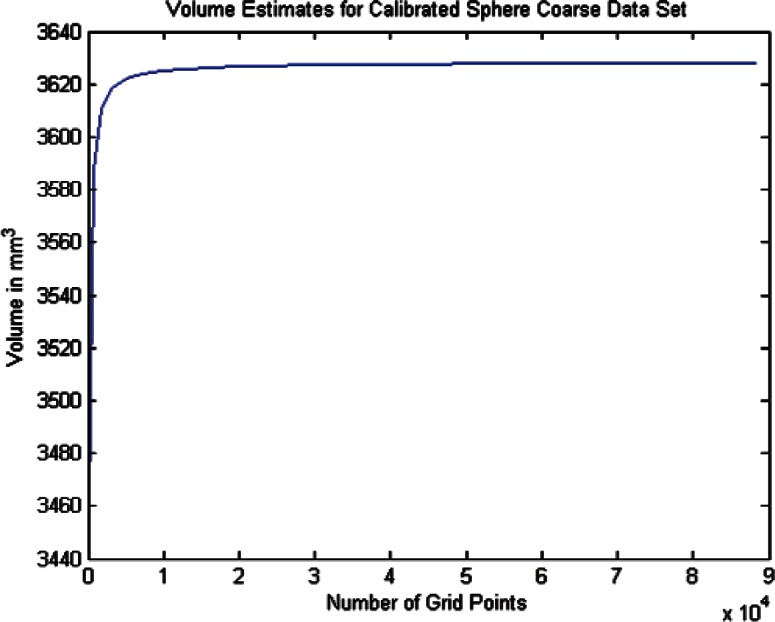
Volume Estimates for Coarse Calibrated Sphere Data Set vs. Grid Size.

For each data set we conducted twenty one estimates of the volumes and volume uncertainties by increasing the density of the surface triangulation. The twenty one were selected since beyond that point the computer time became large and the volume increment per case was minimal. In order to plot the volume results in terms of grid density we use the term grid size by which we mean the product of the number of *θ* values times the number of *ϕ* values for a particular grid density. As shown in Tables 6 through 8 we begin with a grid of ten *θ* and twenty *ϕ* values and compute the volume and uncertainty. The grid size in this 10 × 20 grid is then 200. The *θ* values were then incremented by ten and the *ϕ* values by twenty for each grid case until the last case of 210 *θ* values and 420 *ϕ* values, giving a grid size of 88200 (8.82 × 10^4^). The volumes in Figs. 14 through 19 grow very rapidly and appear to approach a fixed value as the grid size increases. They simply reflect the volume data in the Tables 6 through 8.

#### 6.2.1 Estimating Volume Uncertainties

The uncertainties were estimated by the nonpara-metric “bootstrap” method. It is a computer intensive technique for estimating uncertainties and it involves repeated Monte Carlo resampling from the spherical coordinates of the original measured data sets with radii values modified by the fitting residuals, refitting the model to estimate new volumes, and finally computing an uncertainty for the process from the set of computed volumes. For a full discussion of the bootstrap see Efron and Tibshirani [21] but we will give a brief description here of how we applied the bootstrap method in the current study.

The object of our application of the bootstrap was to develop volume uncertainty as a function of grid size. The process began with the conversion of the Euclidean data points to spherical coordinates. An automatic selection of knots was done. These steps were done once for a given data set. It was assumed that the modifications of the data made during the bootstrap would be small and not affect the knot selection. During the first pass of the bootstrap algorithm the radii of the spherical coordinates were fit by a tensor product of B-splines. The predicted values and residuals from this initial fit were called the master predicted values and master residuals respectively. The computed volume was then put in a list of volumes that was added to in subsequent passes of the algorithm. The algorithm then iterated two hundred times as follows. In the first iteration the master residuals were sampled randomly uniformly with replacement. The resampled residuals were then added to the master predicted radii and a new fit was performed. The new computed volume was added to the volume list and the master residuals sampled randomly uniformly with replacement for the next iteration. The process continued for all two hundred iterations. The standard deviation of the volumes in the volume list was then used as an estimate of the volume uncertainty and the average volume was taken as the reference volume for the chosen grid size. We found that in all cases the expanded uncertainties remained approximately constant and that is why only upper bounds for the expanded uncertainties are reported in the tables below.

#### 6.2.2 Calibrated Sphere Volume Estimates and Uncertainties for the Coarse and Dense Data

As a test of the accuracy of the B-spline volume estimation procedure, we applied the method to the coarse and dense data sets for the calibrated sphere. If the volumes computed by the B-spline method in Table 6 are compared with the sphere fit results in Table 3, we see that the B-spline method and the sphere fit method results differ by approximately 0.01 % for both the coarse and dense data sets. This indicates that the B-spline approach is performing as adequately as the straightforward sphere fit model and thus can be relied upon to produce good results when applied to the phantom data.

#### 6.2.3 Phantom Volume Estimates and Uncertainties for the Coarse Data

**Fig. 16:**
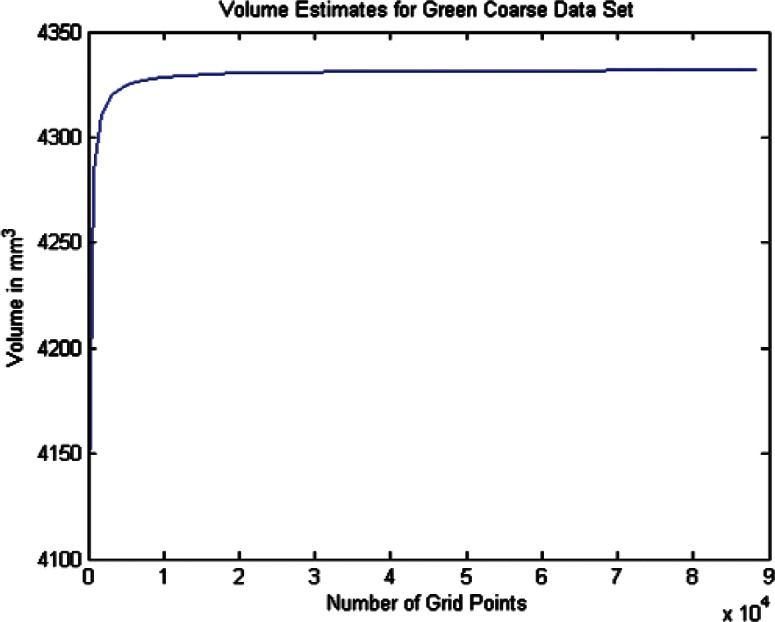
Volume Estimates for Green Coarse Data Set vs. Grid Size.

**Fig. 18:**
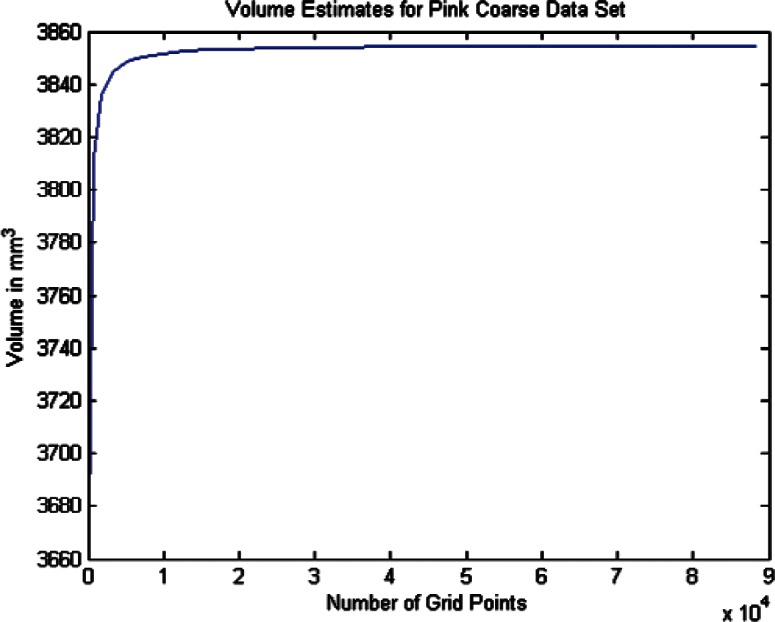
Volume Estimates for Pink Coarse Data Set vs. Grid Size.

In this section we discuss briefly the results of computing the volumes and volume uncertainties for the two phantoms using the coarse data sets. As described earlier, twenty one surface grid cases were used and the results are shown in Tables 7 and 8. Figures 16 and 18 show the trends of the volume estimates as the grid sizes increase. The estimates rise rapidly, and both tend to what appears to be stabilized values. The figures graphically display the values in the tables.

The spherical fit to the Green phantom data in Table 4 indicates an estimated volume of 4331.3 mm^3^. Table 7 indicates an estimated volume of 4331.84 mm^3^ for the largest grid size of 210 *θ* by 420 *ϕ*. This is approximately a 0.01 % difference suggesting that the Green phantom is very nearly spherical.

The spherical fit to the Pink phantom data in Table 5 indicates an estimated volume of 3862.6 mm^3^. Table 8 indicates an estimated volume of 3854.68 mm^3^ for the largest grid size. The difference is approximately 0.21 %, suggesting the slight non-spherical shape of the Pink phantom.

From Tables 7 and 8, the uncertainties are shown bounded on the coarse surface grid by 12 mm^3^ for the Green phantom and 28 mm^3^ for the Pink phantom. This larger uncertainty for the Pink phantom might be due to the non-spherical nature of the Pink phantom. As indicated here, the uncertainties for the Pink phantom are approximately twice those of the Green phantom.

#### 6.2.4 Volume Estimates and Uncertainties for the Dense Data

In this section we report the results of volume estimates and the extended uncertainties for the dense data sets for both phantom surfaces. The first thing of interest is that the stabilized volume estimates for the Green dense data in Table 7 differ from the volume estimate for the Green coarse data by about 0.82 %. This is in the negative direction in that the volume estimates for the Green dense data were smaller than for the Green coarse data. In the case of the volume estimates for the Pink dense data in Table 8, the differences with the volume estimate for the Pink coarse data is approximately 1.33 %. This is in the positive direction in the sense that the volume estimate for the dense data is slightly larger than the volume estimate for the coarse data. These results are consistent with the results of the spherical model fit to the Green and Pink phantoms in Tables 4 and 5. Another thing to notice is that the uncertainties are lower for the dense data sets as shown in Tables 7 through 8 compared to those estimated from the coarse data, although the uncertainty for the Pink phantom is about twice that for the Green phantom. These uncertainties are consistent with the uncertainties computed during the fits of the sphere models in Tables 4 and 5. It is not clear why the results for the dense data differ slightly in the directions they do from the results for the coarse data. A complete analysis of these differences is beyond the scope of this paper, but would be appropriate for a detailed study related to an analysis of the fitting algorithms and their sensitivity to the surface data distribution.

## 7. Summary

The B-spline surface model joined with the Divergence Theorem seems to be a viable approach for estimating volumes of the near-spherical molded phantoms, but the volume results seem to depend strongly on the distribution of the data points on the surface. In the case of sparse data the problem of extending a surface fitted to the data to a grid on the surface leads potentially to unwanted oscillations in the neighborhood of some of the sparse data points. In the current algorithm this problem was dealt with by adding regularization terms to the objective function. These provided a balance between the fit and the surface smoothness in order to control overfitting of the data. The final regularization parameter was *λ* = 0.32 for all data sets.

The results obtained from both the coarse and dense data distributions appear to be consistent with the results from the spherical model fits. As shown in Table 7 the volume estimates for the Green phantom are very close to the estimates obtained by the spherical model in Table 4 suggesting that the Green sphere is essentially spherical in shape. The volume estimates in Table 5 for the Pink sphere are higher by about 0.19 % for the coarse data and 0.15 % for the dense data than the B-spline model estimates in Table 8 suggesting that the Pink phantom might be close to spherical, although it does show some non-spherical tendencies. The uncertainties in the Pink phantom case suggest though that the Pink phantom may indeed have a slightly non-spherical shape. This is also suggested by the difficulty with holding the Pink phantom in the vacuum chuck during one of the surface data probing experiments.

In summary we conclude that the combination of using the CMM to obtain surface data and the B-spline/Divergence Theorem volume estimation method can produce useful results but that there are some limitations. First, the CMM is primarily used for probing manufactured metallic artifacts and its use in probing non-metallic artifacts such as the FDA phantoms can likely lead to some larger uncertainties in volume estimation. Second, the distribution of probed points can lead to results that indicate the models used are sensitive to the point locations. Third, the methods employed may not provide useful volume estimation for more complex artifacts that surely would arise in developing simulated lung cancer phantoms. The current artifacts were near-spherical and even these did not provide fully expected results based on data distribution for the Pink phantom. Further research on the affect of surface data distribution is required. Fourth, the Pole Problem is definitely a limitation that required regularizing of the objective function. Finally, the number of data points obtained on the surfaces was limited by the relative size of the artifact and the CMM probe size.

## Figures and Tables

**Fig. 15:**
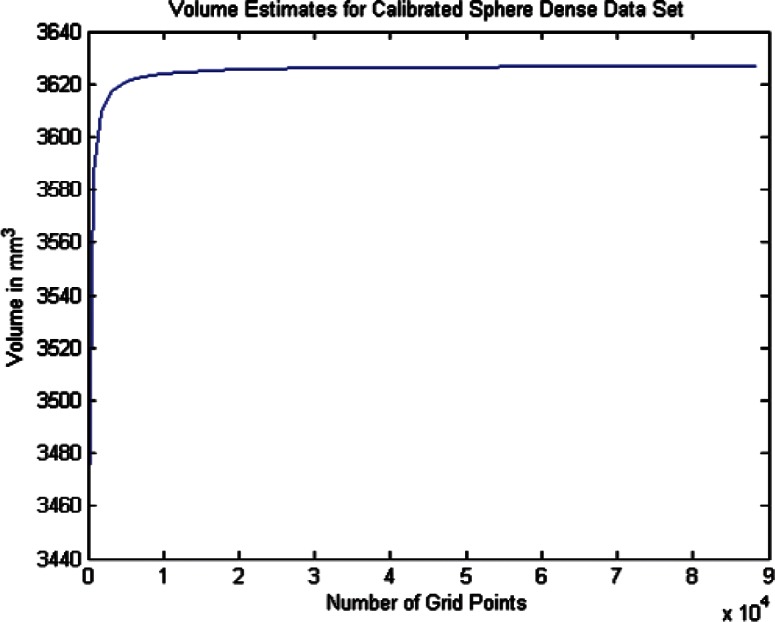
Volume Estimates for Dense Calibrated Sphere Data Set vs. Grid Size.

**Fig. 17:**
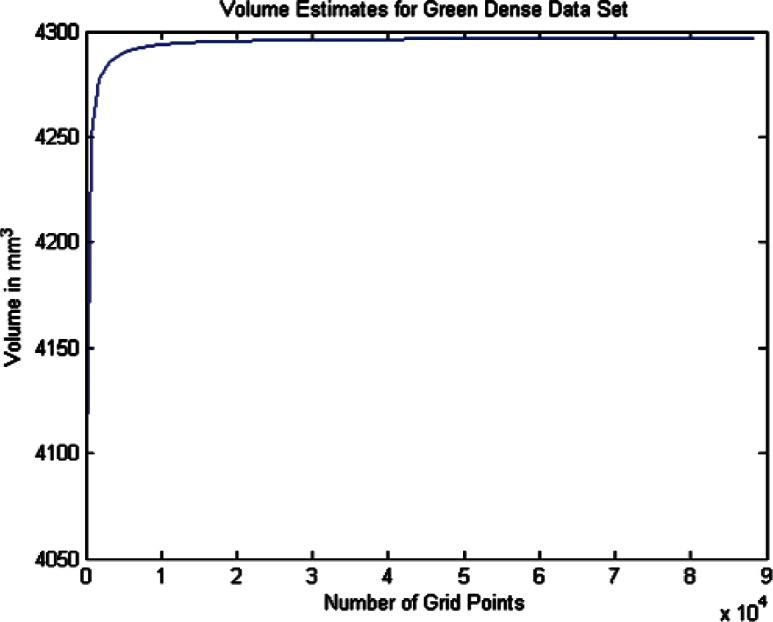
Volume Estimates for Green Dense Data Set vs. Grid Size.

**Fig. 19:**
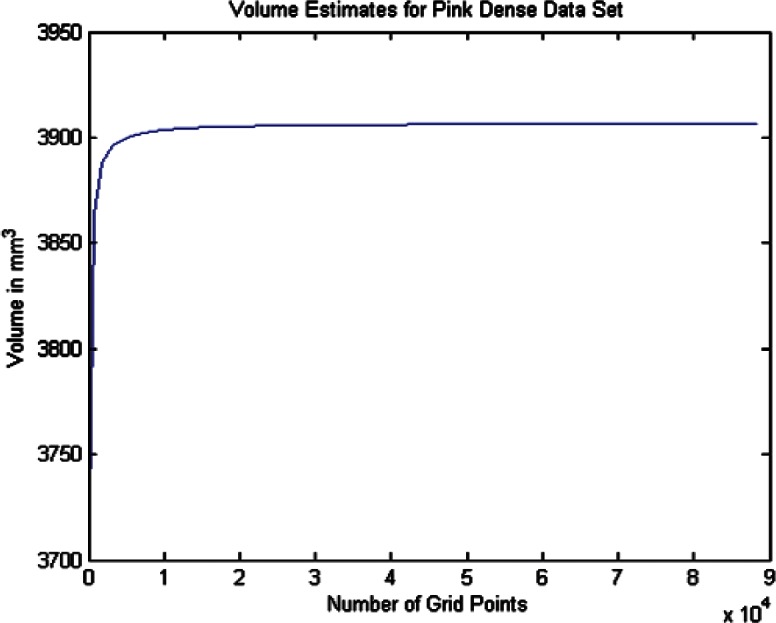
Volume Estimates for Pink Dense Data Set vs. Grid Size.

**Table 1:** Dial Caliper Measurements of Phantoms’ Diameters

	Equatorial	Polar
Pink	20.0 mm	18.4 mm
Green	20.0 mm	20.1 mm

**Table 2:** Octant Equivalence between Euclidean and Spherical Coordinates

Octant Number	3-D Octants to Sperical Coordinates	Spherical Coordinate
	Cartesian Coordinate	
1	*x* ≥ 0, *y* ≥ 0, *z* ≥ 0	0 ≤ *θ* ≤ π/2, 0 ≤ *ϕ* ≤ π/2
2	*x* < 0, *y* ≥ 0, *z* ≥ 0	0 ≤ *θ* ≤ π/2, π/2 ≤ *ϕ* ≤ π
3	*x* < 0, *y* < 0, *z* ≥ 0	0 ≤ *θ* ≤ π/2, π ≤ *ϕ* ≤ 3π/2
4	*x* ≥ 0, *y < 0, z* ≥ 0	0 ≤ *θ* ≤ π/2, 3π/2 ≤ *ϕ* ≤ 2π
5	*x* ≥ 0, *y* ≥ 0, *z* < 0	π/2 ≤ *θ* ≤ π, 0 ≤ *ϕ* ≤ π/2
6	*x* < 0, *y* > 0, *z* < 0	π/2 ≤ *θ* ≤ π, π/2 ≤ *ϕ* ≤ π
7	*x* < 0, *y* < 0, *z* < 0	π/2 ≤ *θ* ≤ π, π ≤ *ϕ* ≤ *3*π/2
8	*x* ≥ 0, *y* < 0, *z* < 0	π/2 ≤ *θ* ≤ π, 3π/2 ≤ *ϕ* ≤ 2π

**Table 3:** Results of a Spherical Fit to Coarse and Dense Data for Calibrated Sphere

Properties	Spherical Fit Results for Calibrated Sphere	Dense
	Coarse	
Center x	0.2803 × 10^−4^	−0.1331 × 10^−2^
Center y	−0.4881 × 10^−3^	−0.2034 × 10^−2^
Center z	−0.6642 × 10^−2^	−0.2564 × 10^−2^
Radius	9.5326	9.5314
Est. Volume	3628.41	3627.14
Mean Rad. Residual	0.3231 × 10^−4^	0.8720 × 10^−4^
Stand. Dev. Rad. Residual	0.2156 × 10^−2^	0.22302 × 10^−2^
Expanded Uncert. Center x	0.2711 × 10^−4^	0.2357 × 10^−4^
Expanded Uncert. Center y	0.2711 × 10^−4^	0.2357 × 10^−4^
Expanded Uncert. Center z	0.4316 × 10^−4^	0.3757 × 10^−4^
Expanded Uncert. Radius	0.2188 × 10^−4^	0.1909 × 10^−4^

**Table 4:** Results of a Spherical Fit to Coarse Data for the Green Phantom

Properties	Spherical Fit Results to Green Phantom	Dense
	Coarse	
Center x	0.8216 × 10^−3^	−0.9172 × 10^−3^
Center y	0.1532 × 10^−2^	0.4275 × 10^−2^
Center z	−0.1832 × 10^−1^	0.1723
Radius	10.1121	10.0850
Est. Volume	4331.3	4296.48
Mean Rad. Residual	−1.4802 × 10^−4^	−0.8450 × 10^−4^
Stand. Dev. Rad. Residual	0.5526 × 10^−1^	0.3093 × 10^−1^
Expanded Uncert. Center x	0.1926 × 10^−1^	0.4757 × 10^−2^
Expanded Uncert. Center y	0.1926 × 10^−1^	0.4753 × 10^−2^
Expanded Uncert. Center z	0.2123 × 10^−1^	0.7261 × 10^−2^
Expanded Uncert. Radius	0.1146 × 10^−1^	0.3632 × 10^−2^

**Table 5:** Results of a Spherical Fit to Coarse Data for the Pink Phantom

Properties	Spherical Fit Results to Pink Phantom	Dense
	Coarse	
Center x	0.7110 × 10^−3^	0.2495 × 10^−1^
Center y	−0.1329 × 10^−2^	0.3272 × 10^−1^
Center z	0.1739 × 10^−3^	0.3719
Radius	9.7332	9.7752
Est. Volume	3862.09	3912.54
Mean Rad. Residual	−0.2315 × 10^−2^	−0.3223 × 10^−3^
Stand. Dev. Rad. Residual	0.2127	0.8935 × 10^−1^
Expanded Uncert. Center x	0.2686	0.3818 × 10^−1^
Expanded Uncert. Center y	0.2693	0.3862 × 10^−1^
Expanded Uncert. Center z	0.2972	0.5832 × 10^−1^
Expanded Uncert. Radius	0.1602	0.2880 × 10^−1^

**Table 6:** Estimated Volumes for Calibrated Sphere Data

Calibrate Sphere Data. Volumes vs Grid Size Expanded Volume Uncertainties: Coarse < 0.6 (mm^3^), Dense < 0.5 (mm^3^)				
Grid Case	θ	*ϕ*	Coarse Volume (mm^3^)	Dense Volume (mm^3^)
1	10	20	3455.05	3453.90
2	20	40	3587.92	3586.74
3	30	60	3610.80	3609.61
4	40	80	3618.63	3617.35
5	50	100	3622.17	3620.88
6	60	120	3624.07	3622.83
7	70	140	3625.21	3623.92
8	80	160	3625.95	3624.66
9	90	180	3626.45	3625.23
10	100	200	3626.81	3625.59
11	110	220	3627.08	3625.86
12	120	240	3627.28	3626.06
13	130	260	3627.43	3626.21
14	140	280	3627.56	3626.34
15	150	300	3627.66	3626.44
16	160	320	3627.74	3626.52
17	170	340	3627.81	3626.59
18	180	360	3627.86	3626.64
19	190	380	3627.91	3626.69
20	200	400	3627.95	3626.73
21	210	420	3627.99	3626.77

**Table 7:** Estimated Volumes for Green Phantom Data

Green Phantom Data. Volumes vs Grid Size Expanded Volume Uncertainties: Coarse < 12 (mm^3^), Dense < 8 (mm^3^)				
Grid Case	θ	*ϕ*	Coarse Volume (mm^3^)	Dense Volume (mm^3^)
1	10	20	4125.34	4092.77
2	20	40	4283.92	4250.85
3	30	60	4310.93	4277.65
4	40	80	4320.67	4285.66
5	50	100	4324.90	4289.86
6	60	120	4327.17	4292.12
7	70	140	4328.53	4293.47
8	80	160	4329.41	4294.35
9	90	180	4330.01	4294.95
10	100	200	4330.44	4295.37
11	110	220	4330.75	4295.69
12	120	240	4330.99	4295.92
13	130	260	4331.07	4296.11
14	140	280	4331.33	4296.26
15	150	300	4331.45	4296.38
16	160	320	4331.54	4296.47
17	170	340	4331.62	4296.55
18	180	360	4331.69	4296.62
19	190	380	4331.75	4296.68
20	200	400	4331.80	4296.72
21	210	420	4331.84	4296.77

**Table 8:** Estimated Volumes for Pink Phantom Data

Pink Phantom Data. Volumes vs Grid Size Expanded Volume Uncertainties: Coarse < 28 (mm^3^), Dense < 18 (mm^3^)				
Grid Case	θ	*ϕ*	Coarse Volume (mm^3^)	Dense Volume (mm^3^)
1	10	20	3668.27	3719.71
2	20	40	3811.59	3863.30
3	30	60	3836.22	3887.99
4	40	80	3844.59	3896.39
5	50	100	3848.41	3900.21
6	60	120	3850.46	3902.27
7	70	140	3851.69	3903.51
8	80	160	3852.48	3904.30
9	90	180	3853.03	3904.85
10	100	200	3853.41	3905.23
11	110	220	3853.70	3905.52
12	120	240	3853.91	3905.74
13	130	260	3854.08	3905.91
14	140	280	3854.22	3906.04
15	150	300	3854.32	3906.15
16	160	320	3854.41	3906.24
17	170	340	3854.48	3906.31
18	180	360	3854.55	3906.37
19	190	380	3854.60	3906.42
20	200	400	3854.64	3906.47
21	210	420	3854.68	3906.50
